# Impact of COVID-19 Infection on Health-Related Quality of Life, Work Productivity and Activity Impairment by Symptom-Based Long COVID Status and Age in the US

**DOI:** 10.3390/healthcare11202790

**Published:** 2023-10-21

**Authors:** Manuela Di Fusco, Joseph C. Cappelleri, Laura Anatale-Tardiff, Henriette Coetzer, Alon Yehoshua, Mary B. Alvarez, Kristen E. Allen, Thomas M. Porter, Laura Puzniak, Ashley S. Cha-Silva, Santiago M. C. Lopez, Xiaowu Sun

**Affiliations:** 1 Pfizer Inc., New York, NY 10001, USAthomas.porter@pfizer.com (T.M.P.);; 2 CVS Health, Woonsocket, RI 02895, USA; laura.anatale-tardiff@bluehealthintelligence.com (L.A.-T.); xiaowu.sun@cvshealth.com (X.S.)

**Keywords:** COVID-19, SARS-CoV-2, quality of life, COVID-19 symptoms, economic, humanistic, long COVID

## Abstract

COVID-19 infection adversely impacts patients’ wellbeing and daily lives. This survey-based study examined differences in patient-reported COVID-19 symptoms, Health-Related Quality of Life (HRQoL) and Work Productivity and Activity Impairment (WPAI) among groups of patients defined based on age and symptom-based long COVID status. Symptomatic, COVID-19-positive US outpatients were recruited from 31 January–30 April 2022. Outcomes were collected via validated instruments at pre-COVID, Day 3, Week 1, Week 4, Month 3 and Month 6 following infection, with changes assessed from pre-COVID and between groups, adjusting for covariates. EQ-5D-5L HRQoL and WPAI scores declined in all groups, especially during the first week. Long COVID patients reported significantly higher symptoms burden and larger drops in HRQoL and WPAI scores than patients without long COVID. Their HRQoL and WPAI scores did not return to levels comparable to pre-COVID through Month 6, except for absenteeism. Patients without long COVID generally recovered between Week 4 and Month 3. Older (>50) and younger adults generally reported comparable symptoms burden and drops in HRQoL and WPAI scores. During the first week of infection, COVID-19-related health issues caused loss of 14 to 26 work hours across the groups. These data further knowledge regarding the differential impacts of COVID-19 on clinically relevant patient groups.

## 1. Introduction

COVID-19 causes a wide variety of acute and long-term symptoms and multi-organ health problems that can persist for weeks or months following infection [1].

There is a rapidly growing body of evidence suggesting that persistent COVID-19 symptoms can negatively impact patients’ Health-Related Quality of Life (HRQoL), productivity and activity levels [2,3,4,5,6]. To date, however, few studies have provided a comprehensive characterization of the impact of COVID-19 on these patient outcomes during the entire course of illness and on the extent to which these differ according to relevant clinical risk factors and patient characteristics.

Studies and literature reviews investigating determinants of HRQoL in adults with COVID-19 have associated certain baseline characteristics with HRQoL, measured using patient-reported EQ-5D tools [7,8,9,10,11]. These include female gender, pre-existing comorbidity and unemployment status. Results for age are mixed, calling for further research: some studies associated younger age with lower HRQoL [7,8], while others reported older age as a determinant of poor HRQoL [9,10,11].

Features of COVID-19 disease have also been associated with diminished HRQoL, including acute disease severity (e.g., ICU admission), not being vaccinated and symptoms burden at the time of infection [7,8,9,10,11]. Although numerous studies have explored the burden of COVID-19 symptoms on HRQoL, most assessed the burden separately for the acute and long COVID phases and compared the HRQoL in long COVID patients with limited control groups [12,13,14,15].

The impacts of a COVID-19 diagnosis on work experience and everyday functioning have been less researched and are still poorly quantified [2,3,4,5,6].

Better understanding of signs, symptoms and magnitude of effects of COVID-19 on productivity, daily activities and HRQoL by patient characteristics and infection features can help healthcare professionals guide prevention and management efforts. Moreover, the data could inform estimates of indirect costs and health utilities in economic evaluations to better understand the economic burden of COVID-19 and the broad value of primary and secondary prevention.

In a prior study, we assessed differences in patient-reported symptoms, HRQoL and Work Productivity and Activity Impairment (WPAI) by vaccination status among 328 adult symptomatic outpatients testing positive for COVID-19 at US community pharmacies and reporting persisting symptoms four weeks following infection. Consistent with the existing body of evidence, we found that pre-infection vaccination status was associated with a lower risk and burden of long COVID symptoms, resulting in better HRQoL and lower WPAI compared to unvaccinated participants [6]. As a continuation of our research efforts on the impact of acute and long COVID on patients’ daily lives, we used this previously described cohort [6] to assess differences in patient-reported outcomes according to age and long COVID status up to 6 months after infection.

The objectives of this study were threefold: (1) to describe characteristics and symptoms of patients by long COVID status and age; (2) to evaluate changes in work productivity, activity levels and HRQoL by long COVID status and age; and (3) to supplement prior research with new quantitative measures of work productivity, expressed as work hours lost and actual hours worked.

## 2. Materials and Methods

### 2.1. Study Design and Participants

The study design has been previously described (clinicaltrials.gov NCT05160636) [6]. Briefly, this was a nationwide prospective Patient-Reported Outcomes (PROs) survey-based study targeting adults at least 18 years of age who tested positive for COVID-19 using Reverse Transcription Polymerase Chain Reaction (RT-PCR) at one of over ~5000 CVS Health test sites across the United States and had self-reported at least one symptom at the time of testing. Patients were eligible for these study analyses if they had symptoms lasting more than four weeks post-acute infection. A total of 328 patients meeting the inclusion and exclusion criteria were selected for this study from a previously described cohort [6]. Recruitment took place between 31 January 2022 and 30 April 2022, with follow-up through 30 October 2022.

### 2.2. Data Sources and Variables

#### 2.2.1. Baseline Characteristics and Acute Symptoms

The baseline characteristics of participants were acquired using the CVS Health pre-test screening questionnaire which comprised self-reported information regarding demographics, comorbidities, COVID-19 vaccination history and social determinants of health, including the Social Vulnerability Index (SVI), and work or residency in a high-risk or healthcare setting. The acute COVID-19 symptoms list from the CDC was utilized to record participant symptoms at the time of testing [1].

#### 2.2.2. Long-Term COVID-19 Symptoms

The study assessed the presence of long COVID symptoms via a questionnaire that included 20 symptoms based on the CDC long COVID symptom list updated in 2022 [1]. The questionnaire was administered beginning at four weeks post-enrollment, with follow-up questionnaires at Months 3 and 6 post-enrollment. In alignment with the CDC definition of long COVID, we considered Week 4 as the start of long-term symptoms [1]. The list of symptoms included general symptoms (tiredness/fatigue, symptoms exacerbated by physical or mental activities, fever, general pain/discomfort), respiratory and cardiac symptoms (difficulty breathing or shortness of breath, cough, chest or stomach pain, heart palpitations), neurological symptoms (change in smell or taste, headache, lightheadedness, “brain fog,” numbness, sleep problems, mood changes, memory loss) and other symptoms (rash, diarrhea, joint or muscle pain, menstrual cycle irregularities).

#### 2.2.3. Exposure Groups

The previously described cohort of 328 participants reporting symptoms at Week 4 following infection [6] was categorized and pre-specified into exposure groups based on their age and long COVID status. They were classified as older adults if they reported being 50 years of age and older at the time of testing and were classified as younger adults if younger than 50 years of age.

Existing studies describing the prevalence of long COVID have employed different thresholds for duration and intensity of symptoms, as well as differing sets of symptoms [16,17,18]. As previously described [6], our study leveraged the clinical case definition of long COVID from the CDC-funded INSPIRE registry, which, as previously reported, employed a similar list and number of symptoms [19,20]. In our main analyses (base case), a patient was classified as having long COVID if reporting ≥3 symptoms at long COVID start (Week 4). In sensitivity analyses, the study used an alternative cutoff threshold of ≥2 symptoms at Week 4.

#### 2.2.4. Health-Related Quality of Life (HRQoL)

Our study assessed HRQoL via the EQ-5D-5L questionnaire [21] that subjects were asked to complete at enrollment, then 1, 3 and 6 months post-enrollment [22]. Five dimensions of EQ-5D-5L at each time point were converted into the Utility Index (UI) using the US-based weights established by Pickard et al. [23]. Lower scores for both EQ VAS and UI correspond to lower overall self-reported health-related quality of life. UI and Visual Analogue Scale (VAS) scores were compared among cohorts and across assessment times [21].

#### 2.2.5. Work Productivity and Activity Impairment

The Work Productivity and Activity Impairment General Health v2.0 (WPAI:GH) measure was used to measure impairments in both paid and unpaid work [24,25]. Participants were asked to complete the survey seven days after presenting for testing and again at Months 1, 3 and 6 post-enrollment. Higher scores correspond to greater activity impairment and work productivity loss. Only employed participants were included for work productivity analyses. WPAI results were compared across cohorts and assessment times.

### 2.3. Statistical Methods

To summarize participant characteristics at baseline and outcomes at follow-up, means and standard deviations for continuous variables and frequency and percentages for categorical variables were used. For between-group differences, *t*-tests and chi-square tests were used to test continuous variables and categorical variables, respectively. When an expected cell frequency was less than 5, Fisher’s exact tests were used for 2-by-2 tables and Fisher–Freeman–Halton tests for r-by-c tables [26,27]. *p*-values were all two-sided.

To estimate the impact of long COVID or age (≥50 vs. <50 years old) on HRQoL and WPAI over time, Mixed Models for Repeated Measures (MMRM) were used [28] with an unstructured covariance matrix for categorical assessment time. For each time point of assessment, Least Squares (LS) mean and standard errors of PRO scores were calculated for each cohort and their difference. The EQ-5D-5L UI and WPAI scores were calculated based on their tool guidelines [21,25]; no imputation was made for missing data and the analyses were based on all available data.

Cohen’s d, or a variation of it, was calculated to examine the difference in pre-COVID scores among patients with or without long COVID or age ≥ 50 or <50 years old, the magnitude of score change from pre-COVID at Week 4, Month 3 and Month 6 within each cohort, as well as the differences between cohorts (with vs. without long COVID; age ≥ 50 vs. <50 years old) [29,30]. Specifically, within-cohort Effect Size (ES) from pre-COVID to follow-up was calculated as mean change scores divided by the standard deviation of change scores [22]. Between-cohort ES was calculated as the difference between cohort means divided by the pooled standard deviation for either pre-COVID scores or change scores from pre-COVID to follow-up [22]. Values of 0.2, 0.5 and 0.8 Standard Deviation (SD) units represent, respectively, “small,” “medium” and “large” effect sizes [22,29].

The study followed the Strengthening the Reporting of Observational Studies in Epidemiology (STROBE) reporting guideline [31]. SAS Version 9.4 (SAS Institute, Cary, NC, USA) was used to conduct all analyses.

## 3. Results

### 3.1. Results by Long COVID Status

#### 3.1.1. Patient Characteristics and Symptoms

As previously reported, among 328 study participants, 130 (39.6%) had long COVID based on the base case definition of ≥3 symptoms at Week 4 [6]. Table 1 reports the baseline characteristics for participants with long COVID (N = 130) and those without it (N = 198). Participants did not differ by vaccination status, age, race, geography, SVI, prior infection status and high-risk setting. Compared with those not reporting long COVID at Week 4, the long COVID cohort was characterized by more females (82.3% vs. 68.2%), a higher proportion of subjects with one or more comorbidities (35.4% vs. 20.7%) and a higher mean number of acute COVID symptoms at the time of infection (6.1 vs. 4.9) (Table 1). Participants with long COVID reported significantly more chills (60.8% vs. 43.4), muscle or body ache (63.1% vs. 51.0%), fatigue (71.5% vs. 56.1%), shortness of breath or difficulty breathing (20.8% vs. 7.6%), congestion or runny nose (83.1% vs. 70.2%), nausea or vomiting (17.7% vs. 9.6%) and diarrhea (26.9% vs. 17.2%) (Table 1). Such characteristics were relatively similar when conducting a sensitivity analysis using a definition of at least two symptoms at Week 4 (Supplemental Table S1).

At long COVID start (Week 4), compared with individuals without COVID-19, subjects with long COVID reported a mean of 6.6 symptoms versus 0.7. The predominant symptoms were tiredness or fatigue (77.7% vs. 17.2%), difficulty thinking or concentrating (59.2% vs. 4.6%), sleep problems (52.3% vs. 6.6%), headache (47.7% vs. 2.5%) and joint or muscle pain (46.9% vs. 2.5%) (Table 2).

Individuals with long COVID continued to experience a higher prevalence of symptoms at Month 3 and Month 6 post-index date compared with individuals without long COVID, with a mean prevalence of 6.4 symptoms versus 0.5 and 6.7 symptoms versus 0.5, respectively. At Month 3, the most predominant symptom was tiredness or fatigue (82.6% vs. 9.8%), followed by difficulty thinking or concentrating (56.9% vs. 4.4%), sleep problems (51.4% vs. 3.8%), headache (39.4% vs. 4.4%) and joint or muscle pain (45.9% vs. 1.6%). At Month 6, the predominant symptom remained tiredness or fatigue (86.8% vs. 13.0%), followed by difficulty thinking or concentrating (56.0% vs. 4.1%), sleep problems (51.6% vs. 3.0%), headache (47.3% vs. 4.1%) and joint or muscle pain (38.5% vs. 1.8%). (Table 2).

#### 3.1.2. EQ-5D-5L

Participants with long COVID reported lower pre-COVID mean EQ VAS and Utility Index (UI) compared with individuals without COVID-19 (84.9 and 0.88 versus 88.6 and 0.94, respectively; Table 3), in line with the observed differences in baseline comorbid status between the two groups. COVID-19 had a detrimental impact on the quality of life of both groups, with the largest ESs for the mean changes from pre-COVID versus soon after infection, at Day 3. At Week 4, patients without long COVID had model-based EQ VAS and UI scores that were numerically lower although comparable to pre-COVID baseline (85.8 and 0.91 versus 88.8 and 0.94, respectively; Table 3). On the other hand, quality of life scores for patients with long COVID did not return to levels comparable to pre-COVID at any point in time. At Month 6, their EQ VAS and UI scores were still significantly lower than pre-COVID (79.3 and 0.80 versus 84.9 and 0.88, respectively; Table 3). The model-based EQ VAS and UI scores in patients with long COVID were significantly lower versus those without long COVID across all time points of the acute (Day 3, Week 4) and long-term (Month 3 and Month 6) survey phases, with medium-to-large ESs (Table 3).

#### 3.1.3. Work Productivity and Activity Impairment

A total of 245 participants (75%) reported being employed at baseline and were eligible to complete the work productivity questions. Of those, 94 (38%) had long COVID and 151 (62%) did not have long COVID.

At enrollment, participants reported pre-COVID mean values for absenteeism, work productivity, missed work hours and actual hours worked that were not significantly different between the two groups. Instead, participants without long COVID reported significantly lower presenteeism than those with long COVID.

COVID-19 had a large impact on all WPAI scores at Week 1 (Table 3). In both groups, the absenteeism levels returned to levels comparable to baseline after Week 4. The presenteeism levels in participants with long COVID continued to be impacted and, at Month 6, did not yet return to levels comparable to baseline. On the other hand, presenteeism levels of participants without long COVID returned to levels comparable to baseline at Week 4.

At enrollment, participants with and without long COVID reported similar pre-COVID baseline mean values of 37 to 38 actual hours worked per week and 4 to 5 missed work hours per week. COVID-19-related health issues were associated with, respectively, 22.3 and 14.3 work hours lost during Week 1 (Table 3). Study participants reported being able to work for, respectively, 15.6 and 21.6 hours during Week 1. The mean work productivity loss at Week 1 was, respectively, 68.0% and 55.0%, corresponding to large ESs in the mean change from pre-COVID baseline and medium ES in the mean changes between the two groups. At Week 4 and Month 6, participants with long COVID did not return to work productivity levels comparable to baseline.

The participants with long COVID reported a pre-COVID mean Activity Impairment (AI) score of 21.3%, significantly higher than those without long COVID (11.1%) (Table 3).

At Week 1, the model-based AI increased to 57.3% and 40.7% in subjects with and without long COVID, corresponding to large ESs in the mean change from pre-COVID baseline and medium ES in the mean changes between the two groups. The AI scores returned to levels comparable to baseline from Week 4 in participants without long COVID. The model-based AI scores for the subjects with long COVID were numerically similar between Week 4 (27.8%), Month 3 (22.5%) and Month 6 (27.9%) and did not return to pre-COVID levels at any point in time.

A summary of observed EQ-5D-5L and WPAI results for those with and without long COVID, with long COVID defined as reporting three or more symptoms, is presented in Supplemental Table S2.

A sensitivity analysis was carried out with long COVID defined as reporting two or more symptoms at Week 4. The results with this alternative definition of long COVID were consistent with the results reported in the main analysis. A summary of observed EQ-5D-5L and WPAI results using the alternative definition is presented in Supplemental Table S3. Model-based LSE estimates are presented in Supplemental Table S4.

### 3.2. Results by Age

#### 3.2.1. Patient Characteristics and Symptoms

Of 328 study participants, 95 were 50 years or older (older adults) and 233 were younger than 50 (younger adults). At enrollment, compared with younger adults, older patients did not differ by vaccination status, race, geography, SVI, prior infection status and number of acute COVID-19 symptoms at the time of infection (Table 4). However, the older adult group was characterized by a higher proportion of males (33.7% vs. 23.2%) and higher comorbidity burden (41.1% vs. 20.6% with at least 1 comorbidity; 0.57 vs. 0.26 mean number of comorbidities). Diabetes and heart conditions or hypertension were significantly more prevalent among older adults. A lower proportion of them reported working in healthcare (5.3% vs. 13.7%) and, in general, in high-risk settings (4.2% vs. 12.4%) than younger adults. Older adults reported a similar number of acute symptoms as younger adults with a mean of 5.2 vs. 5.5, but significantly less fatigue (48.4% vs. 67.8%) (Table 4).

At Week 4, older and younger adults reported a similar mean of, respectively, 3.0 and 3.1 symptoms. The prevalence of symptoms was generally similar across the two age groups, with fatigue being the most prevalent in both groups. Compared with younger adults, older adults experienced less diarrhea (1.2% vs. 8.1%), more joint or muscle pain (31.6% vs. 15.5%) and more cough (37.9% vs. 21.5%) (Table 5).

At Month 3, 81 older adults and 211 younger adults completed the surveys. Both groups reported a mean of 2.7 symptoms, with fatigue persisting as the most prevalent. Compared with younger adults, older adults experienced less diarrhea (1.1% vs. 9.4%) (Table 5).

At Month 6, 71 older adults and 189 younger adults completed the surveys. The groups reported a similar mean of, respectively, 2.5 and 2.7 symptoms. Compared with younger adults, older adults experienced less mood change (5.6% vs. 14.8%), less diarrhea (0.0% vs. 7.4%) and more joint or muscle pain (23.9% vs. 11.1%). Fatigue persisted as most prevalent (Table 5).

#### 3.2.2. EQ-5D-5L

The older and younger adults reported similar pre-COVID mean EQ VAS (86.1 and 87.6, respectively) and mean UI scores (0.92 for both) (Table 6). COVID-19 was associated with a decline in HRQoL in both groups, with the largest ESs for the mean changes between Day 3 and pre-COVID (−12.6 and −14.3 for EQ VAS and −0.11 and −0.14 for UI, respectively). In both age groups, the quality of life scores at all time points were significantly lower than at pre-COVID. 

#### 3.2.3. Work Productivity and Activity Impairment

Of the 245 participants that reported being employed at baseline and were eligible to complete the work productivity questions, 55 (22%) were older adults and 195 (78%) were younger adults.

At enrollment, participants reported pre-COVID mean values for absenteeism, presenteeism, work productivity loss, missed work hours and actual hours worked that were not significantly different by age. Both older and younger adults experienced the largest issues with work activities at Week 1. Compared to pre-COVID baseline, they reported, respectively, that 55.3% and 47.5% of their working time was missed (absenteeism), 36.8% and 35.4% was impaired (presenteeism) and the total work productivity loss was, respectively, 52.2% and 50.0%. Older and younger adults reported, respectively, a total of 25.6 and 22.1 lost work hours during Week 1 of infection, corresponding to a total loss of 20.2 and 18.2 work hours compared to pre-COVID baseline. These work impairments were comparable between the two age groups (Table 6).

Younger adults experienced more long-term impact than older adults, with percentages of work time impaired and total work productivity loss between Week 4 and Month 6 that were generally higher compared to pre-COVID baseline (Table 6).

COVID-19 was associated with large activity impairments during Week 1 across both age groups. The AI scores returned to pre-COVID levels at Month 6 in older adults and at Month 3 among younger adults (Table 6).

A summary of observed EQ-5D-5L and WPAI results by age is presented in Supplemental Table S5. The sensitivity analysis results using the alternative definition of long COVID (two or more symptoms at Week 4) were consistent with the results reported in the base case main analysis.

## 4. Discussion

Evidence on the impacts of a COVID-19 diagnosis and associated health problems on patients’ daily lives is still scarce, especially regarding their work experience. Using a previously described cohort of mild symptomatic patients [6], this study aimed to characterize the trajectory of symptoms and impacts of a COVID-19 diagnosis on HRQoL, activity and work outcomes during the progression of illness up to 6 months after infection, by long COVID status and age.

Our study found that all the four patient groups analyzed (<50 years old, >50 years old, with long COVID, without long COVID) experienced a wide range of symptoms and significant declines in HRQoL. These effects were found to be sustained up to 6 months after infection, regardless of long COVID status and age, as none of the groups returned to levels of wellbeing comparable to pre-COVID, except for participants without on-going long COVID symptoms.

We found that participants with long COVID experienced a significantly greater level of burden than those without long COVID symptoms. Compared with individuals without long COVID, they consistently reported a higher mean number of acute and long-term symptoms throughout the 6-month follow-up period. At every time point, they reported a higher prevalence of tiredness or fatigue, difficulty thinking or concentrating, sleep problems, headache and joint or muscle pain compared to participants without long COVID. Participants with long COVID also consistently reported significantly lower mean EQ-5D VAS and utility values throughout the follow-up.

While comparisons versus existing studies are impaired by differences in study design and methods, we found several similarities and consistencies with the long COVID literature. First, our long COVID cohort had traits that were consistent with studies that investigated predictors of long COVID: female gender, pre-existing comorbidity burden and acute symptoms were significantly more prevalent in the long COVID cohort than in the cohort of participants without long COVID [1,2,3,4,5,32]. Second, our symptoms burden results are in line with prior research on long COVID that reported fatigue as the most prevalent and pervasive symptom [1,2,3,4,5,7,13,33]. Third, our comparative results for symptoms burden and HRQoL detriments are directionally in line with a study conducted in Japan [15] that employed a relatively similar study design. The author team compared symptoms burden and HRQoL (measured with EQ-5D-3L) between long COVID patients and patients with a history of COVID-19 and no on-going symptoms. Consistent with our study, the authors reported that participants with long COVID reported more prolonged symptoms and an overall greater symptoms burden that was expressed with significantly lower mean EQ VAS and utility values. Additional studies assessing HRQoL detriments in long COVID patients compared outcomes versus normative or no controls, hindering side-by-side comparisons [12,13,14,15].

Literature investigating determinants of HRQoL in adults with COVID-19 using patient-reported EQ-5D tools have reported mixed results for age [7,8,9,10,11]. Some studies associated younger age with lower HRQoL [7,8], while others reported older age as a determinant of low HRQoL [9,10,11]. In contrast with existing literature, in our study, younger and older adults reported comparable symptoms burden. The prevalence of symptoms was generally similar between older and younger adults, with fatigue being the most prevalent in both groups and with older adults experiencing less diarrhea and more joint or muscle pain over the follow-up period. The two age groups also reported similar declines in HRQoL. While symptom prevalence and HRQoL estimates vary across studies due to heterogeneous definitions, settings and methods, we observed that the magnitude of decline in HRQoL scores during the acute phase was similar to existing research [33], although the evolution over time was found to differ, most likely related to differences in patient characteristics and study design. A longitudinal cohort study in the Belgian adult population testing positive for COVID-19 reported a mean UI before infection of 0.92 (95% CI = 0.917; 0.923) regardless of long COVID status [33]. The mean UI was 0.80 (95% CI = 0.795; 0.805) at the time of infection, with the drop greater than the minimal detectable change threshold and considered clinically meaningful [33]. In our study, the pre-COVID UI was 0.92 in the two age groups (regardless of long COVID status) and dropped to 0.80 in older adults and 0.78 in younger adults. However, in the Belgian population the UI increased to 0.91 (95% CI = 0.905; 0.911) after 3 months [33], while at Month 3 in our study the UI increased less prominently to 0.82 in older adults and 0.87 in younger adults and was still significantly lower than pre-COVID.

Our study found that COVID-19 deeply affected patients’ work experience during the course of illness and is among the first to provide quantitative measures of work hours lost. The largest work impairments were experienced at the time of infection during Week 1, with lingering long-term effects on presenteeism and work productivity in participants with long COVID and younger adults. Long COVID patients were estimated to miss 28.6 hours of work during Week 1 of infection, corresponding to a decline of 23.9 hours versus pre-COVID. Participants without long COVID were also affected, with 19.3 missed hours of work during Week 1, corresponding to a mean change of 15.4 hours versus pre-COVID. Older and younger adults were similarly impacted at Week 1 with a loss of 20.2 and 18.2 working hours, respectively. Further analyses of Week 1 work hours lost revealed that unvaccinated participants were the most impacted, with the highest number of work hours lost (mean: 28.5). Those boosted with BNT162b2 had the lowest work hours lost (mean: 15.9), solidifying prior evidence of broad benefits of COVID-19 vaccination with BNT162b2 on patient-centric outcomes (Supplemental Table S6) [6].

COVID-19 was associated with large activity impairments during Week 1 across all groups. The activity impairment scores did not return to pre-COVID levels at any point in time among long COVID patients and more slowly for older adults (Month 6) than younger adults (Month 3).

Although our results on work experience and activity levels are not directly comparable to prior survey-based studies due to the difference in study design, our data similarly suggest that COVID-19 can deeply affect paid and unpaid work. O’Mahoney et al. [8] found that a high proportion of long COVID patients reported moderate or severe limitations in their ability to carry out daily activities, with over a third of their sample reporting severe impairments in their ability to work. Similarly, Davis et al. reported that a quarter of the long COVID sample in their study were unable to work due to illness, and almost half required reduced work schedules compared to pre-COVID. Our results complement and supplement these findings by providing different quantitative measures of work productivity losses and activity impairments. Our study showed that a COVID-19 diagnosis had large impacts across age and long COVID status. Long COVID participants were significantly more impacted than those without long COVID, while older and younger adults generally experienced comparable burden. These data suggest that a significant number of people will experience prolonged symptoms that will impact quality of life and functional capacity. The findings provide further evidence that both COVID-19 and long COVID are important public health issues that could affect adults of all ages and may have longer-term effects even after recovery from the acute infection.

To our knowledge, our study is among the first to provide a detailed characterization of the impact of COVID-19 and its evolution throughout the duration of illness according to long COVID status and age [2,3,4,5,6]. It is also among the first to report work hours lost and actual hours worked using validated patient-reported outcome measures, which allows further use to inform estimates of indirect costs.

The study is subject to several limitations. As previously described [6], the self-reported nature of the study means that data may be subject to missingness, errors, recall bias, social desirability bias and selection bias associated with attrition. By Month 6, 21% of the 328 participants were lost to follow-up, which could be due to response fatigue and/or survey burden. Our study population only included adults, was over-represented by females and the source population from which subjects were enrolled was relatively healthy. The WPAI analyses had a smaller eligible population and analyses were impacted by relatively small sample sizes, especially for older adults.

We used a long COVID definition based on presence of symptoms and did not assess severity of symptoms. A diverse set of descriptors exists based on number, type and duration of long COVID symptoms and conditions [18], and a universal definition of long COVID has not been established yet. While we conducted a sensitivity analysis using an alternative long COVID definition, our long COVID results may not be fully comparable with existing research. Despite adjusting for several covariates in the model, there is still a risk of residual confounding. These findings may not be generalizable to populations that were excluded from the study or to prior or future variants, other countries or time periods. We did not analyze outcomes based on different SARS-CoV-2 variants.

This study contributes to knowledge gaps related to patient-reported outcomes of COVID-19. Characterization of acute infection and long COVID continues to evolve, and future studies could corroborate these findings with different data collection methods and designs. Moreover, our analyses focused on two variables that have been shown to affect the evolution of COVID-19 patient outcomes (long COVID status and age). Stratification analyses by vaccination status were previously reported for this cohort [6] and further solidified the growing evidence that COVID-19 vaccines could alleviate the detrimental effects of COVID-19 analyzed in this study. Future research could assess these outcomes in patient cohorts defined by additional clinical risk factors, socio-demographic characteristics and treatment history.

## 5. Conclusions

This study characterized the differential impacts of COVID-19 infection on patient-reported symptoms, HRQoL and WPAI by age and long COVID status. All the patient groups analyzed experienced significant symptoms burden and declines in HRQoL and WPAI scores during the course of their infection. Participants with long COVID experienced a significantly greater level of burden than those with a history of COVID-19 infection and no on-going long COVID symptoms. However, younger and older adults generally reported comparable symptoms burden and drops in HRQoL and WPAI scores. During the first week of infection, COVID-19-related health issues caused loss of between 14 and 26 work hours across the groups.

These data reaffirm that long COVID is a central public health issue requiring continuous research, monitoring and medical management. They also highlight that COVID-19 can significantly affect daily lives of people of all ages, in both the acute and long-term phase, further reinforcing the need for broad access to prevention and management efforts regardless of age. Finally, the new quantitative measures of productivity supplement prior research and could inform estimates of indirect costs in economic evaluations supporting public health policy.

## Figures and Tables

**Table 1:** Patient Characteristics and acute symptoms experienced by patients with long COVID vs. those without long COVID, at long COVID start (Week 4).

	All	With Long COVID	No Long COVID	*p*-Value
Total, n (%)	328	130	198	
Index vaccination status ^a^				0.091
Boosted	87 (26.5%)	26 (20.0%)	61 (30.8%)	
Primed	86 (26.2%)	36 (27.7%)	50 (25.3%)	
Unvaccinated	155 (47.3%)	68 (52.3%)	87 (43.9%)	
Age, years				
Mean, SD	42.0 (14.5)	41.7 (13.9)	42.2 (14.9)	0.774
18–29	73 (22.3%)	29 (22.3%)	44 (22.2%)	0.536
30–49	160 (48.8%)	63 (48.5%)	97 (49.0%)	
50–64	67 (20.4%)	30 (23.1%)	37 (18.7%)	
≥65	28 (8.5%)	8 (6.2%)	20 (10.1%)	
Gender				0.004
Female	242 (73.8%)	107 (82.3%)	135 (68.2%)	
Male	86 (26.2%)	23 (17.7%)	63 (31.8%)	
Race/Ethnicity				0.698
White or Caucasian (not Hispanic or Latino)	234 (71.3%)	96 (73.8%)	138 (69.7%)	
Black or African American	13 (4.0%)	6 (4.6%)	7 (3.5%)	
Hispanic	44 (13.4%)	13 (10.0%)	31 (15.7%)	
Asian	16 (4.9%)	6 (4.6%)	10 (5.1%)	
Patient refused	9 (2.7%)	3 (2.3%)	6 (3.0%)	
Other	12 (3.7%)	6 (4.6%)	6 (3.0%)	
CMS geographic region (n, %)				0.935
Region 1: ME, NH, VT, MA, CT, RI	15 (4.6%)	6 (4.6%)	9 (4.6%)	
Region 2: NY, NJ, PR, VI	9 (2.7%)	4 (3.1%)	5 (2.5%)	
Region 3: PA, DE, MD, DC, WV, VA	31 (9.5%)	11 (8.5%)	20 (10.1%)	
Region 4: KY, TN, NC, SC, GA, MS, AL, FL	116 (35.4%)	51 (39.2%)	65 (32.8%)	
Region 5: MN, WI, IL, MI, IN, OH	47 (14.3%)	19 (14.6%)	28 (14.1%)	
Region 6: NM, OK, AR, TX, LA	59 (18.0%)	20 (15.4%)	39 (19.7%)	
Region 7: NE, IA, KS, MO	16 (4.9%)	7 (5.4%)	9 (4.6%)	
Region 8: MT, ND, SD, WY, UT, CO	1 (0.3%)	0 (0.0%)	1 (0.5%)	
Region 9: CA, NV, AZ, GU	33 (10.1%)	12 (9.2%)	21 (10.6%)	
Region 10: AK, WA, OR, ID	1 (0.3%)	0 (0.0%)	1 (0.5%)	
US geographic region				0.810
Northeast	41 (12.5%)	15 (11.5%)	26 (13.1%)	
South	188 (57.3%)	77 (59.2%)	111 (56.1%)	
Midwest	63 (19.2%)	26 (20.0%)	37 (18.7%)	
West	36 (11.0%)	12 (9.2%)	24 (12.1%)	
Previously tested positive	121 (36.9%)	51 (39.2%)	70 (35.4%)	0.477
Work in healthcare	37 (11.3%)	14 (10.8%)	23 (11.6%)	0.813
Work in high-risk setting	33 (10.1%)	18 (13.8%)	15 (7.6%)	0.065
Live in high-risk setting	16 (4.9%)	10 (7.7%)	6 (3.0%)	0.055
Social vulnerability index ^b^, mean (SD)	0.43 (0.22)	0.46 (0.22)	0.42 (0.21)	0.102
Self-reported comorbidity				
Number of comorbidities, mean (SD)	0.35 (0.65)	0.46 (0.72)	0.28 (0.59)	0.012
Asthma or chronic lung disease	30 (9.2%)	18 (13.8%)	12 (6.1%)	0.017
Cirrhosis of the liver	1 (0.3%)	1 (0.8%)	0 (0.0%)	0.217
Immunocompromised conditions or weakened immune system ^c^	16 (4.9%)	7 (5.4%)	9 (4.6%)	0.730
Diabetes	11 (3.4%)	6 (4.6%)	5 (2.5%)	0.304
Heart conditions or hypertension	41 (12.5%)	20 (15.4%)	21 (10.6%)	0.201
Overweight or obesity	16 (4.9%)	8 (6.2%)	8 (4.0%)	0.385
At least 1 comorbidity	87 (26.5%)	46 (35.4%)	41 (20.7%)	0.003
Index day ^d^ acute COVID-19 symptoms				
Number of acute COVID-19 symptoms, mean (SD)	5.39 (2.57)	6.13 (2.50)	4.90 (2.50)	<0.001
Systemic symptoms				
Fever	127 (38.7%)	53 (40.8%)	74 (37.4%)	0.537
Chills	165 (50.3%)	79 (60.8%)	86 (43.4%)	0.002
Muscle or body aches	183 (55.8%)	82 (63.1%)	101 (51.0%)	0.031
Headache	224 (68.3%)	99 (76.2%)	125 (63.1%)	0.013
Fatigue	204 (62.2%)	93 (71.5%)	111 (56.1%)	0.005
Respiratory symptoms				
Shortness of breath or difficulty breathing	42 (12.8%)	27 (20.8%)	15 (7.6%)	0.001
Cough	243 (74.1%)	99 (76.2%)	144 (72.7%)	0.489
Sore throat	187 (57.0%)	80 (61.5%)	107 (54.0%)	0.180
New/Recent loss of taste or smell	35 (10.7%)	19 (14.6%)	16 (8.1%)	0.061
Congestion or runny nose	247 (75.3%)	108 (83.1%)	139 (70.2%)	0.008
GI symptoms				
Nausea or vomiting	42 (12.8%)	23 (17.7%)	19 (9.6%)	0.032
Diarrhea	69 (21.0%)	35 (26.9%)	34 (17.2%)	0.034

SD: standard deviation; ^a^ Definitions in Di Fusco et al. (2023) [2,3,4,5,6]; ^b^ The Social Vulnerability Index uses 16 US census variables to help local officials identify communities that may need support before, during or after disasters; ^c^ Immunocompromised conditions include compromised immune system (such as from immunocompromising drugs, solid organ or blood stem cell transplant, HIV or other conditions), conditions that result in a weakened immune system, including cancer treatment, and kidney failure or end stage renal disease; ^d^ COVID-19 test nasal swab day.

**Table 2:** Summary of Post-COVID symptoms in patients with long COVID vs. those without long COVID, at long COVID start (Week 4), at Month 3 and at Month 6.

	Week 4			Month 3			Month 6		
	With Long COVID	No Long COVID	*p*-Value	With Long COVID	No Long COVID	*p*-Value	With Long COVID	No Long COVID	*p*-Value
N of patients	130	198		109	183		91	169	
Number of symptoms, mean (SD)	6.6 (3.3)	0.7 (0.8)	<0.001	6.4 (3.3)	0.5 (0.8)	<0.001	6.7 (3.5)	0.5 (0.7)	<0.001
≥1 General symptom	116 (89.2%)	38 (19.2%)	<0.001	99 (90.8%)	22 (12.0%)	<0.001	89 (97.8%)	23 (13.6%)	<0.001
Tiredness or fatigue	101 (77.7%)	34 (17.2%)	<0.001	90 (82.6%)	18 (9.8%)	<0.001	79 (86.8%)	22 (13.0%)	<0.001
Symptoms that get worse after physical or mental activities	49 (37.7%)	1 (0.5%)	<0.001	34 (31.2%)	2 (1.1%)	<0.001	26 (28.6%)	2 (1.2%)	<0.001
Fever	1 (0.8%)	0 (0.0%)	1.000	1 (0.9%)	0 (0.0%)	0.3733	3 (3.3%)	0 (0.0%)	0.042
General pain/discomfort	47 (36.2%)	3 (1.5%)	<0.001	41 (37.6%)	2 (1.1%)	<0.001	36 (39.6%)	0 (0.0%)	<0.001
≥1 Respiratory and cardiac symptom	100 (76.9%)	42 (21.2%)	<0.001	66 (60.6%)	17 (9.3%)	<0.001	59 (64.8%)	9 (5.3%)	<0.001
Difficulty breathing or shortness of breath	48 (36.9%)	10 (5.1%)	<0.001	35 (32.1%)	7 (3.8%)	<0.001	29 (31.9%)	3 (1.8%)	<0.001
Cough	56 (43.1%)	30 (15.2%)	<0.001	29 (26.6%)	9 (4.9%)	<0.001	27 (29.7%)	5 (3.0%)	<0.001
Chest or stomach pain	30 (23.1%)	2 (1.0%)	<0.001	17 (15.6%)	0 (0.0%)	<0.001	17 (18.7%)	0 (0.0%)	<0.001
Fast-beating or pounding heart (also known as heart palpitations)	36 (27.7%)	2 (1.0%)	<0.001	27 (24.8%)	2 (1.1%)	<0.001	28 (30.8%)	1 (0.6%)	<0.001
≥1 Neurologic symptom	121 (93.1%)	40 (20.2%)	<0.001	104 (95.4%)	36 (19.7%)	<0.001	90 (98.9%)	31 (18.3%)	<0.001
Change in smell or taste	43 (33.1%)	8 (4.0%)	<0.001	30 (27.5%)	5 (2.7%)	<0.001	24 (26.4%)	3 (1.8%)	<0.001
Headache	62 (47.7%)	5 (2.5%)	<0.001	43 (39.4%)	8 (4.4%)	<0.001	43 (47.3%)	7 (4.1%)	<0.001
Dizziness on standing (lightheadedness)	42 (32.3%)	3 (1.5%)	<0.001	38 (34.9%)	5 (2.7%)	<0.001	28 (30.8%)	10 (5.9%)	<0.001
Difficulty thinking or concentrating (sometimes referred to as “brain fog”)	77 (59.2%)	9 (4.6%)	<0.001	62 (56.9%)	8 (4.4%)	<0.001	51 (56.0%)	7 (4.1%)	<0.001
Pins-and-needles feeling	23 (17.7%)	1 (0.5%)	<0.001	25 (22.9%)	3 (1.6%)	<0.001	26 (28.6%)	1 (0.6%)	<0.001
Sleep problems	68 (52.3%)	13 (6.6%)	<0.001	56 (51.4%)	7 (3.8%)	<0.001	47 (51.6%)	5 (3.0%)	<0.001
Mood changes	35 (26.9%)	1 (0.5%)	<0.001	30 (27.5%)	1 (0.6%)	<0.001	32 (35.2%)	0 (0.0%)	<0.001
Memory loss	36 (27.7%)	2 (1.0%)	<0.001	33 (30.3%)	2 (1.1%)	<0.001	35 (38.5%)	2 (1.2%)	<0.001
≥1 Other symptom	86 (66.2%)	20 (10.1%)	<0.001	75 (68.8%)	13 (7.1%)	<0.001	63 (69.2%)	13 (7.7%)	<0.001
Diarrhea	21 (16.2%)	2 (1.0%)	<0.001	17 (15.6%)	1 (0.6%)	<0.001	13 (14.3%)	1 (0.6%)	<0.001
Joint or muscle pain	61 (46.9%)	5 (2.5%)	<0.001	50 (45.9%)	3 (1.6%)	<0.001	35 (38.5%)	3 (1.8%)	<0.001
Rash	10 (7.7%)	1 (0.5%)	<0.001	7 (6.4%)	2 (1.1%)	0.015	5 (5.5%)	0 (0.0%)	0.005
Changes in period cycles	16 (15.0%)	12 (8.9%)	0.143	29 (30.9%)	7 (5.8%)	<0.001	23 (29.9%)	9 (7.9%)	<0.001

**Table 3:** Least-Square Estimates of HRQoL and WPAI for patients with long COVID and those without long COVID ^a^.

	Patients with Long COVID ^b^				Patients without Long COVID				Between-Cohort Difference: With and without Long COVID		
	Mean Score	Mean Change from Pre-COVID			Mean Score	Mean Change from Pre-COVID					
	LSE (95% CI)	LSE (95% CI)	*p*-Value	ES	LSE (95% CI)	LSE (95% CI)	*p*-Value	ES	LSE (95% CI)	*p*-Value	ES
EQ VAS											
Pre-COVID ^c^	84.9 (12.2)				88.6 (10.2)				−3.7 (11.0)	0.004	−0.33
Day 3 ^d^	70.3 (67.1, 73.5)	−17.1 (−20.3, −13.9)	<0.001	−1.05	77.3 (74.6, 80.1)	−10.0 (−12.8, −7.3)	<0.001	−0.83	−7.0 (−10.2, −3.8)	<0.001	−0.41
Week 4	79.2 (76.5, 82.0)	−8.1 (−10.8, −5.4)	<0.001	−0.52	85.8 (83.3, 88.2)	−1.6 (−4.0, 0.9)	0.204	−0.20	−6.5 (−8.7, −4.3)	<0.001	−0.48
Month 3	80.9 (78.1, 83.7)	−6.5 (−9.3, −3.7)	<0.001	−0.43	85.5 (83.0, 88.0)	−1.8 (−4.4, 0.7)	0.148	−0.20	−4.7 (−7.0, −2.3)	<0.001	−0.34
Month 6	79.3 (76.3, 82.4)	−8.0 (−11.1, −5.0)	<0.001	−0.48	86.4 (83.8, 89.0)	−1.0 (−3.6, 1.6)	0.461	−0.11	−7.0 (−9.8, −4.3)	<0.001	−0.52
EQ-5D-5L Utility Index (US weights)											
Pre-COVID ^c^	0.88 (0.14)				0.94 (0.10)				−0.06 (0.12)	<0.001	−0.50
Day 3 ^d^	0.72 (0.68, 0.76)	−0.20 (−0.24, −0.16)	<0.001	−0.84	0.85 (0.82, 0.89)	−0.07 (−0.10, −0.03)	<0.001	−0.58	−0.14 (−0.17, −0.10)	<0.001	−0.68
Week 4	0.81 (0.78, 0.84)	−0.11 (−0.14, −0.08)	<0.001	−0.69	0.91 (0.88, 0.94)	−0.01 (−0.04, 0.02)	0.673	−0.07	−0.10 (−0.13, −0.08)	<0.001	−0.72
Month 3	0.82 (0.79, 0.86)	−0.10 (−0.13, −0.06)	<0.001	−0.51	0.89 (0.86, 0.93)	−0.03 (−0.06, 0.01)	0.112	−0.21	−0.07 (−0.10, −0.04)	<0.001	−0.40
Month 6	0.80 (0.77, 0.84)	−0.12 (−0.15, −0.08)	<0.001	−0.55	0.90 (0.86, 0.93)	−0.02 (−0.06, 0.01)	0.142	−0.24	−0.09 (−0.12, −0.06)	<0.001	−0.53
WPAI GH											
Absenteeism											
Pre-COVID ^c^	7.6 (21.2)				7.4 (21.6)				0.2 (21.4)	0.943	0.01
Week 1 ^d^	62.9 (54.6, 71.1)	56.0 (47.8, 64.3)	<0.001	1.47	48.9 (42.2, 55.6)	42.0 (35.3, 48.7)	<0.001	1.09	14.0 (4.6, 23.4)	0.004	0.37
Week 4	5.0 (0.5, 9.5)	−1.9 (−6.4, 2.6)	0.409	−0.08	4.2 (0.2, 8.3)	−2.7 (−6.7, 1.4)	0.201	−0.11	0.8 (−2.7, 4.2)	0.663	0.05
Month 3	8.1 (3.1, 13.1)	1.2 (−3.8, 6.2)	0.636	0.04	6.9 (2.4, 11.5)	0.1 (−4.4, 4.6)	0.977	0.00	1.1 (−3.1, 5.4)	0.598	0.06
Month 6	8.4 (3.1, 13.7)	1.5 (−3.8, 6.8)	0.573	0.05	3.8 (−0.6, 8.3)	−3.0 (−7.5, 1.4)	0.179	−0.14	4.5 (−0.3, 9.4)	0.067	0.29
Presenteeism											
Pre-COVID ^c^	13.2 (22.6)				7.6 (16.5)				5.6 (19.1)	0.028	0.29
Week 1 ^d^	49.7 (41.1, 58.3)	40.0 (31.4, 48.6)	<0.001	1.27	38.5 (31.6, 45.4)	28.9 (21.9, 35.8)	<0.001	0.91	11.1 (2.3, 20.0)	0.014	0.39
Week 4	19.7 (13.8, 25.5)	10.0 (4.2, 15.9)	0.001	0.34	3.8 (−1.6, 9.2)	−5.9 (−11.3, −0.5)	0.032	−0.29	15.9 (11.4, 20.4)	<0.001	0.86
Month 3	15.4 (8.2, 22.6)	5.7 (−1.4, 12.9)	0.116	0.19	8.4 (2.4, 14.5)	−1.2 (−7.3, 4.8)	0.692	−0.05	7.0 (0.4, 13.5)	0.037	0.31
Month 6	23.6 (16.9, 30.3)	13.9 (7.2, 20.6)	<0.001	0.40	5.5 (−0.3, 11.2)	−4.2 (−9.9, 1.5)	0.151	−0.21	18.1 (12.3, 23.9)	<0.001	1.02
Work productivity loss											
Pre-COVID ^c^	15.1 (24.7)				11.5 (21.5)				3.6 (22.8)	0.236	0.16
Week 1 ^d^	68.0 (58.8, 77.1)	55.8 (46.6, 64.9)	<0.001	1.75	55.0 (47.6, 62.3)	42.8 (35.4, 50.2)	<0.001	1.19	13.0 (3.7, 22.2)	0.006	0.43
Week 4	22.2 (15.8, 28.6)	10.0 (3.6, 16.4)	0.002	0.30	4.4 (−1.5, 10.3)	−7.8 (−13.7, −1.9)	0.010	−0.31	17.8 (13.0, 22.6)	<0.001	0.91
Month 3	17.4 (9.5, 25.3)	5.2 (−2.6, 13.1)	0.191	0.15	10.9 (4.2, 17.5)	−1.3 (−8.0, 5.4)	0.702	−0.05	6.5 (−0.6, 13.7)	0.074	0.27
Month 6	26.0 (18.6, 33.3)	13.8 (6.4, 21.2)	<0.001	0.36	7.5 (1.2, 13.8)	−4.7 (−10.9, 1.6)	0.143	−0.18	18.5 (12.1, 24.8)	<0.001	0.93
Activity impairment											
Pre-COVID ^c^	21.3 (29.3)				11.1 (20.8)				10.2 (24.5)	<0.001	0.42
Week 1 ^d^	57.3 (51.1, 63.5)	42.4 (36.2, 48.6)	<0.001	1.17	40.7 (35.4, 45.9)	25.8 (20.5, 31.0)	<0.001	0.80	16.6 (10.3, 23.0)	<0.001	0.57
Week 4	27.8 (22.8, 32.8)	12.9 (7.9, 17.9)	<0.001	0.36	7.9 (3.5, 12.3)	−7.0 (−11.4, −2.6)	0.002	−0.30	19.9 (15.6, 24.2)	<0.001	0.93
Month 3	22.5 (16.9, 28.1)	7.6 (2.0, 13.2)	0.008	0.21	12.5 (7.7, 17.3)	−2.4 (−7.2, 2.5)	0.336	−0.10	10.0 (4.7, 15.2)	<0.001	0.41
Month 6	27.9 (22.3, 33.5)	13.0 (7.5, 18.6)	<0.001	0.33	8.1 (3.5, 12.8)	−6.7 (−11.4, −2.1)	0.005	−0.30	19.8 (14.5, 25.0)	<0.001	0.93
Hours missed due to health											
Pre-COVID ^c^	4.7 (15.1)				3.9 (11.8)				0.8 (13.2)	0.654	0.06
Week 1 ^d^	26.2 (22.5, 30.0)	22.3 (18.5, 26.1)	<0.001	1.08	18.2 (15.2, 21.3)	14.3 (11.3, 17.3)	<0.001	0.82	8.0 (3.6, 12.4)	<0.001	0.44
Week 4	1.9 (0.1, 3.7)	−2.1 (−3.9, −0.3)	0.024	−0.13	0.7 (−0.9, 2.3)	−3.3 (−4.9, −1.6)	<0.001	−0.28	1.2 (−0.3, 2.7)	0.127	0.20
Month 3	2.3 (0.1, 4.4)	−1.7 (−3.8, 0.5)	0.125	−0.09	1.8 (−0.1, 3.7)	−2.1 (−4.0, −0.3)	0.023	−0.17	0.5 (−1.6, 2.5)	0.650	0.06
Month 6	1.5 (−0.6, 3.6)	−2.4 (−4.5, −0.3)	0.024	−0.15	1.1 (−0.6, 2.8)	−2.8 (−4.5, −1.1)	0.001	−0.22	0.4 (−1.6, 2.3)	0.697	0.06
Actual hours worked											
Pre-COVID ^c^	36.7 (13.5)				37.8 (14.6)				−1.1 (14.2)	0.557	−0.08
Week 1 ^d^	15.6 (11.3, 20.0)	−21.6 (−26.0, −17.3)	<0.001	−1.24	21.6 (17.9, 25.3)	−15.7 (−19.4, −12.0)	<0.001	−0.85	−6.0 (−10.1, −1.9)	0.005	−0.34
Week 4	34.1 (30.2, 37.9)	−3.2 (−7.1, 0.6)	0.101	−0.20	35.3 (31.9, 38.8)	−2.0 (−5.4, 1.5)	0.261	−0.14	−1.3 (−4.6, 2.1)	0.459	−0.09
Month 3	34.4 (30.0, 38.8)	−2.9 (−7.3, 1.5)	0.195	−0.14	33.6 (29.8, 37.3)	−3.7 (−7.5, 0.0)	0.050	−0.23	0.8 (−3.2, 4.9)	0.683	0.06
Month 6	32.0 (27.3, 36.7)	−5.3 (−10.0, −0.6)	0.029	−0.31	32.9 (29.2, 36.7)	−4.3 (−8.1, −0.6)	0.024	−0.26	−0.9 (−5.5, 3.6)	0.684	−0.06

LSE = least-square estimate; CI = confidence interval; ES = effect size. ^a^ Multivariate models include variables for number of symptoms ≥ 3 (yes, no), time and interaction of number of symptoms ≥ 3 by time, vaccination status and interaction of time by vaccination status, as well as covariates of participant pre-COVID-19 symptom onset score, sociodemographic characteristics (age, sex, regions, social vulnerability, race/ethnicity, high-risk occupations), previously tested positive for COVID-19, severity of acute illness (number of symptoms reported on index date) and immunocompromised status. ^b^ Long COVID was defined as ≥3 symptoms reported. ^c^ At pre-COVID, scores were summarized for observed measures with mean (SD). ^d^ Measures on Day 3 and Week 1 were summarized based on number of post-COVID symptoms at Week 4.

**Table 4:** Patient Characteristics and acute symptoms experienced by subjects aged 50 years or older or younger than 50, at long COVID start (Week 4).

	All	Age ≥ 50	Age < 50	*p*-Value
Total, n (%)	328	95	233	
Index vaccination status				0.157
Boosted	87 (26.5%)	29 (30.5%)	58 (24.9%)	
Primed	86 (26.2%)	29 (30.5%)	57 (24.5%)	
Unvaccinated	155 (47.3%)	37 (38.9%)	118 (50.6%)	
Age, years				
Mean, SD	42.0 (14.5)	61.0 (8.2)	34.2 (7.8)	<0.001
18–29	73 (22.3%)		73 (31.3%)	
30–49	160 (48.8%)		160 (68.7%)	
50–64	67 (20.4%)	67 (70.5%)		
≥65	28 (8.5%)	28 (29.5%)		
Gender				0.050
Female	242 (73.8%)	63 (66.3%)	179 (76.8%)	
Male	86 (26.2%)	32 (33.7%)	54 (23.2%)	
Race/Ethnicity				0.069
White or Caucasian (not Hispanic or Latino)	234 (71.3%)	76 (80.0%)	158 (67.8%)	
Black or African American	13 (4.0%)	4 (4.2%)	9 (3.9%)	
Hispanic	44 (13.4%)	7 (7.4%)	37 (15.9%)	
Asian	16 (4.9%)	1 (1.1%)	15 (6.4%)	
Patient refused	9 (2.7%)	4 (4.2%)	5 (2.2%)	
Other	12 (3.7%)	3 (3.2%)	9 (3.9%)	
CMS geographic region (n, %)				0.305
Region 1: ME, NH, VT, MA, CT, RI	15 (4.6%)	3 (3.2%)	12 (5.2%)	
Region 2: NY, NJ, PR, VI	9 (2.7%)	4 (4.2%)	5 (2.2%)	
Region 3: PA, DE, MD, DC, WV, VA	31 (9.5%)	6 (6.3%)	25 (10.7%)	
Region 4: KY, TN, NC, SC, GA, MS, AL, FL	116 (35.4%)	40 (42.1%)	76 (32.6%)	
Region 5: MN, WI, IL, MI, IN, OH	47 (14.3%)	12 (12.6%)	35 (15.0%)	
Region 6: NM, OK, AR, TX, LA	59 (18.0%)	20 (21.1%)	39 (16.7%)	
Region 7: NE, IA, KS, MO	16 (4.9%)	1 (1.1%)	15 (6.4%)	
Region 8: MT, ND, SD, WY, UT, CO	1 (0.3%)	0 (0.0%)	1 (0.4%)	
Region 9: CA, NV, AZ, GU	33 (10.1%)	9 (9.5%)	24 (10.3%)	
Region 10: AK, WA, OR, ID	1 (0.3%)	0 (0.0%)	1 (0.4%)	
US geographic region				0.070
Northeast	63 (19.2%)	13 (13.7%)	50 (21.5%)	
South	41 (12.5%)	8 (8.4%)	33 (14.2%)	
Midwest	188 (57.3%)	65 (68.4%)	123 (52.8%)	
West	36 (11.0%)	9 (9.5%)	27 (11.6%)	
Previously tested positive	121 (36.9%)	38 (40.0%)	83 (35.6%)	0.456
Work in healthcare	37 (11.3%)	5 (5.3%)	32 (13.7%)	0.028
Work in high-risk setting	33 (10.1%)	4 (4.2%)	29 (12.4%)	0.025
Live in high-risk setting	16 (4.9%)	5 (5.3%)	11 (4.7%)	
Social vulnerability index ^a^, mean (SD)	0.43 (0.22)	0.44 (0.19)	0.43 (0.22)	0.741
Self-reported comorbidity				
Number of comorbidities, mean (SD)	0.35 (0.65)	0.57 (0.81)	0.26 (0.55)	<0.001
Asthma or chronic lung disease	30 (9.2%)	12 (12.6%)	18 (7.7%)	0.162
Cirrhosis of the liver	1 (0.3%)	1 (1.1%)	0 (0.0%)	0.117
Immunocompromised conditions or weakened immune system ^b^	16 (4.9%)	5 (5.3%)	11 (4.7%)	0.836
Diabetes	11 (3.4%)	8 (8.4%)	3 (1.3%)	0.001
Heart conditions or hypertension	41 (12.5%)	22 (23.2%)	19 (8.2%)	<0.001
Overweight or obesity	16 (4.9%)	6 (6.3%)	10 (4.3%)	0.440
At least 1 comorbidity	87 (26.5%)	39 (41.1%)	48 (20.6%)	<0.001
Index day ^c^ acute COVID-19 symptoms				
Number of acute COVID-19 symptoms, mean (SD)	5.4 (2.6)	5.2 (2.5)	5.5 (2.6)	0.392
Systemic symptoms				
Fever	127 (38.7%)	32 (33.7%)	95 (40.8%)	0.232
Chills	165 (50.3%)	51 (53.7%)	114 (48.9%)	0.434
Muscle or body aches	183 (55.8%)	51 (53.7%)	132 (56.7%)	0.623
Headache	224 (68.3%)	60 (63.2%)	164 (70.4%)	0.202
Fatigue	204 (62.2%)	46 (48.4%)	158 (67.8%)	0.001
Respiratory symptoms				
Shortness of breath or difficulty breathing	42 (12.8%)	10 (10.5%)	32 (13.7%)	0.430
Cough	243 (74.1%)	76 (80.0%)	167 (71.7%)	0.119
Sore throat	187 (57.0%)	53 (55.8%)	134 (57.5%)	0.775
New/Recent loss of taste or smell	35 (10.7%)	12 (12.6%)	23 (9.9%)	0.463
Congestion or runny nose	247 (75.3%)	74 (77.9%)	173 (74.2%)	0.487
GI symptoms				
Nausea or vomiting	42 (12.8%)	8 (8.4%)	34 (14.6%)	0.129
Diarrhea	69 (21.0%)	21 (22.1%)	48 (20.6%)	0.762

SD: standard deviation; ^a^ The Social Vulnerability Index uses 16 US census variables to help local officials identify communities that may need support before, during or after disasters; ^b^ Immunocompromised conditions include compromised immune system (such as from immunocompromising drugs, solid organ or blood stem cell transplant, HIV or other conditions), conditions that result in a weakened immune system, including cancer treatment, and kidney failure or end stage renal disease; ^c^ COVID-19 test nasal swab day.

**Table 5:** Summary of Post-COVID symptoms in patients aged 50 years or older or younger than 50, at long COVID start (Week 4), at Month 3 and at Month 6, n(%).

	Week 4			Month 3			Month 6		
	Age ≥ 50	Age < 50	*p*-Value ^a^	Age ≥ 50	Age < 50	*p*-Value ^a^	Age ≥ 50	Age < 50	*p*-Value ^a^
N of patients	95	233		81	211		71	189	
Number of symptoms, mean (SD)	3.0 (3.4)	3.1 (3.7)	0.884	2.7 (3.5)	2.7 (3.6)	0.972	2.5 (3.5)	2.7 (3.7)	0.626
≥2 symptoms	52 (54.7%)	123 (52.8%)	0.749	41 (50.6%)	96 (45.5%)	0.433	33 (46.5%)	82 (43.4%)	0.655
≥3 symptoms	38 (40.0%)	92 (39.5%)	0.931	32 (39.5%)	77 (36.5%)	0.634	22 (31.0%)	69 (36.5%)	0.406
≥1 General symptom	44 (46.3%)	110 (47.2%)	0.883	36 (44.4%)	85 (40.3%)	0.518	30 (42.3%)	82 (43.4%)	0.870
Tiredness or fatigue	38 (40.0%)	97 (41.6%)	0.785	29 (35.8%)	79 (37.4%)	0.795	25 (35.2%)	76 (40.2%)	0.461
Symptoms that get worse after physical or mental activities	13 (13.7%)	37 (15.9%)	0.616	10 (12.3%)	26 (12.3%)	0.996	9 (12.7%)	19 (10.1%)	0.543
Fever	1 (1.1%)	0 (0.0%)	0.117	0 (0.0%)	1 (0.5%)	0.535	0 (0.0%)	3 (1.6%)	0.286
General pain/discomfort	17 (17.9%)	33 (14.2%)	0.394	17 (21.0%)	26 (12.3%)	0.061	11 (15.5%)	25 (13.2%)	0.638
≥1 Respiratory and cardiac symptom	45 (47.4%)	97 (41.6%)	0.342	25 (30.9%)	58 (27.5%)	0.567	16 (22.5%)	52 (27.5%)	0.416
Difficulty breathing or shortness of breath	12 (12.6%)	46 (19.7%)	0.126	12 (14.8%)	30 (14.2%)	0.897	9 (12.7%)	23 (12.2%)	0.912
Cough	36 (37.9%)	50 (21.5%)	0.002	14 (17.3%)	24 (11.4%)	0.179	12 (16.9%)	20 (10.6%)	0.167
Chest or stomach pain	7 (7.4%)	25 (10.7%)	0.352	4 (4.9%)	13 (6.2%)	0.690	3 (4.2%)	14 (7.4%)	0.355
Fast-beating or pounding heart (also known as heart palpitations)	12 (12.6%)	26 (11.2%)	0.705	9 (11.1%)	20 (9.5%)	0.676	6 (8.5%)	23 (12.2%)	0.396
≥1 Neurologic symptom	49 (51.6%)	112 (48.1%)	0.564	42 (51.9%)	98 (46.4%)	0.408	36 (50.7%)	85 (45.0%)	0.409
Change in smell or taste	18 (18.9%)	33 (14.2%)	0.278	8 (9.9%)	27 (12.8%)	0.492	6 (8.5%)	21 (11.1%)	0.531
Headache	16 (16.8%)	51 (21.9%)	0.304	14 (17.3%)	37 (17.5%)	0.960	12 (16.9%)	38 (20.1%)	0.559
Dizziness on standing (lightheadedness)	13 (13.7%)	32 (13.7%)	0.991	13 (16.0%)	30 (14.2%)	0.693	8 (11.3%)	30 (15.9%)	0.349
Difficulty thinking or concentrating (sometimes referred to as “brain fog”)	25 (26.3%)	61 (26.2%)	0.980	17 (21.0%)	53 (25.1%)	0.459	17 (23.9%)	41 (21.7%)	0.698
Pins-and-needles feeling	5 (5.3%)	19 (8.2%)	0.362	11 (13.6%)	17 (8.1%)	0.151	9 (12.7%)	18 (9.5%)	0.458
Sleep problems	20 (21.1%)	61 (26.2%)	0.329	22 (27.2%)	41 (19.4%)	0.151	16 (22.5%)	36 (19.0%)	0.531
Mood changes	9 (9.5%)	27 (11.6%)	0.579	7 (8.6%)	24 (11.4%)	0.497	4 (5.6%)	28 (14.8%)	0.045
Memory loss	12 (12.6%)	26 (11.2%)	0.705	9 (11.1%)	26 (12.3%)	0.775	10 (14.1%)	27 (14.3%)	0.967
≥1 Other symptom	31 (32.6%)	75 (32.2%)	0.938	22 (27.2%)	66 (31.3%)	0.492	18 (25.4%)	58 (30.7%)	0.399
Diarrhea	1 (1.1%)	22 (9.4%)	0.007	1 (1.2%)	17 (8.1%)	0.030	0 (0.0%)	14 (7.4%)	0.018
Joint or muscle pain	30 (31.6%)	36 (15.5%)	0.001	18 (22.2%)	35 (16.6%)	0.263	17 (23.9%)	21 (11.1%)	0.009
Rash	2 (2.1%)	9 (3.9%)	0.423	3 (3.7%)	6 (2.8%)	0.703	1 (1.4%)	4 (2.1%)	0.711
Changes in period cycles	0 (0.0%)	28 (15.6%)		1 (1.9%)	35 (21.6%)		0 (0.0%)	32 (22.1%)	

SD: standard deviation; ^a^ *P* values of *t*-test for number of symptoms, Chi-square tests or Fisher’s exact tests when any one cell has an expected frequency less than 5 for individual symptoms and number of symptom category comparing age ≥ 50 and age < 50 years.

**Table 6:** Least-Square Estimates of HRQoL and WPAI for patients aged 50 years or older and younger than 50 ^a^.

	Age ≥ 50 Years				Age < 50 Years				Between-Cohort Difference: Age ≥ 50 Years and <50 Years		
	Mean Score	Mean Change from Pre-COVID			Mean Score	Mean Change from Pre-COVID					
	LSE (95% CI)	LSE (95% CI)	*p*-Value	ES	LSE (95% CI)	LSE (95% CI)	*p*-Value	ES	LSE (95% CI)	*p*-Value	ES
EQ VAS											
Pre-COVID ^b^	86.1 (12.7)				87.6 (10.5)				−1.5 (11.2)	0.282	−0.13
Day 3 ^c^	75.1 (71.5, 78.7)	−12.2 (−15.8, −8.6)	<0.001	−0.82	74.6 (71.9, 77.3)	−12.8 (−15.5, −10.0)	<0.001	−0.91	0.5 (−3.0, 4.1)	0.767	0.03
Week 4	81.6 (78.3, 84.9)	−5.8 (−9.0, −2.5)	0.001	−0.42	84.2 (81.7, 86.8)	−3.1 (−5.7, −0.6)	0.016	−0.27	−2.6 (−5.7, 0.4)	0.088	−0.18
Month 3	82.2 (78.9, 85.6)	−5.1 (−8.5, −1.8)	0.003	−0.43	84.7 (82.1, 87.2)	−2.7 (−5.2, −0.2)	0.038	−0.22	−2.4 (−5.5, 0.6)	0.121	−0.17
Month 6	83.7 (80.1, 87.2)	−3.7 (−7.3, −0.1)	0.043	−0.28	84.5 (81.8, 87.1)	−2.9 (−5.5, −0.3)	0.031	−0.23	−0.8 (−4.2, 2.6)	0.646	−0.05
EQ-5D-5L Utility Index (US weights)											
Pre-COVID ^b^	0.92 (0.12)				0.92 (0.12)				0.00 (0.12)	0.864	0.02
Day 3 ^c^	0.82 (0.77, 0.87)	−0.10 (−0.15, −0.05)	<0.001	−0.66	0.80 (0.77, 0.84)	−0.12 (−0.15, −0.08)	<0.001	−0.57	0.01 (−0.03, 0.06)	0.532	0.07
Week 4	0.84 (0.80, 0.88)	−0.08 (−0.12, −0.04)	<0.001	−0.59	0.90 (0.87, 0.93)	−0.02 (−0.05, 0.01)	0.195	−0.15	−0.05 (−0.09, −0.02)	0.002	−0.32
Month 3	0.84 (0.79, 0.88)	−0.08 (−0.13, −0.04)	<0.001	−0.48	0.89 (0.86, 0.93)	−0.03 (−0.06, 0.01)	0.135	−0.16	−0.06 (−0.10, −0.02)	0.008	−0.29
Month 6	0.85 (0.80, 0.89)	−0.07 (−0.12, −0.02)	0.003	−0.49	0.89 (0.85, 0.92)	−0.03 (−0.07, 0.00)	0.049	−0.20	−0.04 (−0.08, 0.01)	0.097	−0.18
WPAI GH											
Absenteeism											
Pre-COVID ^b^	8.0 (20.5)				7.3 (21.7)				0.7 (21.4)	0.821	0.03
Week 1 ^c^	61.1 (50.8, 71.3)	54.2 (43.9, 64.5)	<0.001	1.40	51.4 (45.4, 57.5)	44.6 (38.5, 50.6)	<0.001	1.13	9.6 (−1.4, 20.7)	0.087	0.25
Week 4	9.0 (3.8, 14.1)	2.1 (−3.1, 7.3)	0.428	0.08	2.6 (−1.1, 6.2)	−4.3 (−8.0, −0.7)	0.021	−0.18	6.4 (1.6, 11.2)	0.009	0.42
Month 3	9.3 (3.0, 15.6)	2.4 (−3.9, 8.7)	0.449	0.11	6.1 (2.1, 10.2)	−0.7 (−4.8, 3.3)	0.724	−0.03	3.1 (−3.0, 9.3)	0.312	0.18
Month 6	4.5 (−1.4, 10.4)	−2.4 (−8.3, 3.5)	0.423	−0.13	4.9 (1.0, 8.8)	−2.0 (−5.9, 1.9)	0.312	−0.08	−0.4 (−5.9, 5.1)	0.891	−0.02
Presenteeism											
Pre-COVID ^b^	10.7 (22.1)				9.4 (18.3)				1.3 (19.2)	0.654	0.07
Week 1 ^c^	44.3 (33.8, 54.7)	34.6 (24.2, 45.0)	<0.001	1.07	43.4 (37.0, 49.8)	33.7 (27.3, 40.1)	<0.001	1.06	0.9 (−9.7, 11.5)	0.873	0.03
Week 4	12.1 (4.9, 19.3)	2.4 (−4.8, 9.6)	0.507	0.11	10.5 (5.4, 15.6)	0.9 (−4.2, 6.0)	0.733	0.03	1.5 (−4.8, 7.9)	0.635	0.07
Month 3	13.0 (4.5, 21.6)	3.4 (−5.2, 11.9)	0.439	0.17	12.1 (6.6, 17.6)	2.4 (−3.1, 8.0)	0.385	0.09	0.9 (−7.1, 8.9)	0.822	0.04
Month 6	12.7 (4.5, 20.8)	3.0 (−5.1, 11.2)	0.467	0.11	11.9 (6.4, 17.5)	2.3 (−3.3, 7.8)	0.424	0.08	0.8 (−6.9, 8.4)	0.846	0.04
Work productivity loss											
Pre-COVID ^b^	16.0 (25.9)				12.0 (21.8)				4.0 (22.8)	0.257	0.18
Week 1 ^c^	61.8 (50.9, 72.8)	49.7 (38.7, 60.6)	<0.001	1.39	59.9 (53.0, 66.8)	47.7 (40.8, 54.6)	<0.001	1.37	1.9 (−9.1, 13.0)	0.729	0.06
Week 4	13.4 (5.4, 21.3)	1.2 (−6.7, 9.1)	0.767	0.04	11.4 (5.8, 17.1)	−0.8 (−6.4, 4.9)	0.791	−0.03	2.0 (−5.1, 9.0)	0.583	0.09
Month 3	14.8 (5.3, 24.2)	2.6 (−6.8, 12.0)	0.586	0.12	14.2 (8.0, 20.3)	2.0 (−4.1, 8.2)	0.517	0.06	0.6 (−8.2, 9.4)	0.896	0.02
Month 6	16.5 (7.7, 25.4)	4.4 (−4.5, 13.2)	0.332	0.14	13.1 (7.0, 19.2)	1.0 (−5.1, 7.0)	0.756	0.03	3.4 (−4.8, 11.7)	0.417	0.15
Activity impairment											
Pre-COVID ^b^	17.5 (26.7)				14.2 (24.2)				3.3 (25.0)	0.283	0.13
Week 1 ^c^	47.2 (39.9, 54.5)	32.3 (25.1, 39.6)	<0.001	0.96	45.9 (40.4, 51.4)	31.0 (25.6, 36.5)	<0.001	0.89	1.3 (−5.9, 8.5)	0.720	0.04
Week 4	20.1 (13.7, 26.5)	5.2 (−1.2, 11.6)	0.110	0.16	12.5 (7.5, 17.4)	−2.4 (−7.4, 2.5)	0.336	−0.08	7.6 (1.8, 13.5)	0.011	0.30
Month 3	22.2 (15.4, 28.9)	7.3 (0.5, 14.1)	0.035	0.29	12.7 (7.6, 17.8)	−2.2 (−7.3, 2.9)	0.395	−0.07	9.5 (3.1, 15.8)	0.004	0.37
Month 6	18.0 (11.1, 25.0)	3.1 (−3.8, 10.1)	0.375	0.10	12.4 (7.2, 17.5)	−2.5 (−7.6, 2.6)	0.332	−0.08	5.7 (−0.9, 12.2)	0.091	0.22
Hours missed due to health											
Pre-COVID ^b^	5.4 (13.5)				3.9 (13.1)				1.5 (13.2)	0.460	0.11
Week 1 ^c^	24.5 (19.7, 29.3)	20.5 (15.7, 25.4)	<0.001	0.87	20.5 (17.7, 23.3)	16.6 (13.7, 19.4)	<0.001	0.94	4.0 (−1.3, 9.3)	0.138	0.22
Week 4	2.1 (0.1, 4.2)	−1.8 (−3.9, 0.2)	0.078	−0.13	1.1 (−0.4, 2.5)	−2.9 (−4.3, −1.4)	<0.001	−0.21	1.0 (−0.8, 2.9)	0.277	0.18
Month 3	1.6 (−1.0, 4.1)	−2.4 (−5.0, 0.2)	0.068	−0.24	2.4 (0.7, 4.1)	−1.5 (−3.2, 0.1)	0.070	−0.09	−0.9 (−3.4, 1.7)	0.510	−0.11
Month 6	1.7 (−0.6, 4.0)	−2.2 (−4.5, 0.1)	0.061	−0.15	1.4 (−0.1, 2.9)	−2.5 (−4.1, −1.0)	0.001	−0.19	0.3 (−1.8, 2.5)	0.759	0.05
Actual hours worked											
Pre-COVID ^b^	39.4 (14.8)				36.8 (14.0)				2.5 (14.2)	0.237	0.18
Week 1 ^c^	18.5 (13.6, 23.4)	−18.8 (−23.7, −13.9)	<0.001	−0.92	20.9 (17.6, 24.1)	−16.4 (−19.7, −13.2)	<0.001	−0.93	−2.4 (−7.2, 2.5)	0.336	−0.13
Week 4	34.8 (30.6, 39.1)	−2.4 (−6.7, 1.8)	0.260	−0.14	36.1 (33.1, 39.1)	−1.2 (−4.2, 1.8)	0.433	−0.08	−1.3 (−5.3, 2.7)	0.536	−0.09
Month 3	31.2 (26.2, 36.3)	−6.1 (−11.1, −1.0)	0.019	−0.29	35.8 (32.6, 39.1)	−1.5 (−4.7, 1.8)	0.378	−0.09	−4.6 (−9.6, 0.3)	0.066	−0.31
Month 6	36.0 (30.7, 41.3)	−1.3 (−6.5, 4.0)	0.638	−0.08	32.9 (29.6, 36.3)	−4.4 (−7.7, −1.0)	0.010	−0.26	3.1 (−2.1, 8.4)	0.245	0.20

LSE = least-square estimate; CI = confidence interval; ES = effect size. ^a^ Multivariate models include variables for number of symptoms ≥ 3, time and interaction of number of symptom ≥ 3 by time, vaccination status and interaction of time by vaccination status, as well as covariates of participant pre-COVID-19 symptom onset score, sociodemographic characteristics (age, sex, regions, social vulnerability, race/ethnicity, high-risk occupations), previously tested positive for COVID-19, severity of acute illness (number of symptoms reported on index date) and immunocompromised status. ^b^ Pre-COVID, scores were summarized for observed measures with mean (SD). ^c^ Measures on Day 3 and Week 1 were summarized based on number of post-COVID symptoms at Week 4.

## Data Availability

Aggregated data that support the findings of this study are available upon reasonable request from the corresponding author M.D.F., subject to review. These data are not publicly available due to them containing information that could compromise research participant privacy/consent.

## Supplementary Materials

The following supporting information can be downloaded at: https://www.mdpi.com/article/10.3390/healthcare11202790/s1, Supplemental Table S1. Patient Characteristics and acute symptoms experienced by subjects with long COVID vs. those without long COVID, at long COVID start (Week 4), with sensitivity analysis long COVID definition (≥2 symptoms); Supplemental Table S2. Summary of HRQoL and WPAI results for those with long COVID and those without long COVID, with base case long COVID definition (≥3 symptoms); Supplemental Table S3. Summary of HRQoL and WPAI results for those with long COVID and those without long COVID, with sensitivity analysis long COVID definition (≥2 symptoms); Supplemental Table S4. Least-Square Estimates of HRQoL and WPAI results for those with long COVID and those without long COVID, with sensitivity analysis long COVID definition (≥2 symptoms); Supplemental Table S5. Summary of HRQoL and WPAI results for patients age ≥ 50 and age < 50 years; Supplemental Table S6. Work hours lost at Week 1 by vaccination status.

## Abbreviations

CDC: Centers for Disease Control and Prevention; CI: confidence interval; EQ-5D-5L: EuroQoL Group 5 dimension and 5 level questionnaires; ES: effect size; GH: general health; HRQoL: health-related quality of life; MMRMs: mixed models for repeated measures; SD: standard deviation; SE: standard error; STROBE: Strengthening the Reporting of Observational Studies in Epidemiology; UI: utility index; VAS: visual analogue scale; WPAI: work productivity and impairment.
