# Supplementary material for: Impact of COVID-19 Infection on Health-Related Quality of Life, Work Productivity and Activity Impairment by Symptom-Based Long COVID Status and Age in the US
Source: Healthcare (Basel). 2023 Oct 21;11(20):2790. doi: 10.3390/healthcare11202790 (PMC10606451; doi:10.3390/healthcare11202790)
Supplement: Supplementary file 1 [file healthcare-11-02790-s001.zip › healthcare-2603567-supplementary.pdf]

Supplemental Table S1. Patient Characteristics and acute symptoms experienced by subjects with long COVID vs those without long COVID, at long COVID start (week 4), with sensitive analysis long COVID definition (≥ 2 symptoms)

	**All**	**With Long COVID**	**Without Long COVID**	***p*-Value**
Total, n (%)	328	175	153	
Index vaccination status [a]				0.397
Boosted	87 (26.5%)	41 (23.4%)	46 (30.1%)	
Primed	86 (26.2%)	48 (27.4%)	38 (24.8%)	
Unvaccinated	155 (47.3%)	86 (49.1%)	69 (45.1%)	
Age, years				
Mean, SD	42.0 (14.5)	42.1 (13.9)	41.8 (15.2)	0.869
18-29	73 (22.3%)	38 (21.7%)	35 (22.9%)	0.498
30-49	160 (48.8%)	85 (48.6%)	75 (49.0%)	
50-64	67 (20.4%)	40 (22.9%)	27 (17.6%)	
≥65	28 (8.5%)	12 (6.9%)	16 (10.5%)	
Gender				0.025
Female	242 (73.8%)	138 (78.9%)	104 (68.0%)	
Male	86 (26.2%)	37 (21.1%)	49 (32.0%)	
Race / Ethnicity				0.837
White or Caucasian (not Hispanic or Latino)	234 (71.3%)	125 (71.4%)	109 (71.2%)	
Black or African American	13 (4.0%)	9 (5.1%)	4 (2.6%)	
Hispanic	44 (13.4%)	21 (12.0%)	23 (15.0%)	
Asian	16 (4.9%)	8 (4.6%)	8 (5.2%)	
Patient Refused	9 (2.7%)	5 (2.9%)	4 (2.6%)	
Other	12 (3.7%)	7 (4.0%)	5 (3.3%)	
CMS Geographic Region (n, %)				0.825
Region 1: ME, NH, VT, MA, CT, RI	15 (4.6%)	8 (4.6%)	7 (4.6%)	
Region 2: NY, NJ, PR, VI	9 (2.7%)	5 (2.9%)	4 (2.6%)	
Region 3: PA, DE, MD, DC, WV, VA	31 (9.5%)	15 (8.6%)	16 (10.5%)	

	All	With Long COVID	Without Long COVID	*p*-Value
Region 4: KY, TN, NC, SC, GA, MS, AL, FL	116 (35.4%)	68 (38.9%)	48 (31.4%)	
Region 5: MN, WI, IL, MI, IN, OH	47 (14.3%)	25 (14.3%)	22 (14.4%)	
Region 6: NM, OK, AR, TX, LA	59 (18.0%)	27 (15.4%)	32 (20.9%)	
Region 7: NE, IA, KS, MO	16 (4.9%)	9 (5.1%)	7 (4.6%)	
Region 8: MT, ND, SD, WY, UT, CO	1 (0.3%)	1 (0.6%)	0 (0.0%)	
Region 9: CA, NV, AZ, GU	33 (10.1%)	17 (9.7%)	16 (10.5%)	
Region 10: AK, WA, OR, ID	1 (0.3%)	0 (0.0%)	1 (0.7%)	
US Geographic Region				0.880
Northeast	41 (12.5%)	20 (11.4%)	21 (13.7%)	
South	188 (57.3%)	103 (58.9%)	85 (55.6%)	
Midwest	63 (19.2%)	34 (19.4%)	29 (19.0%)	
West	36 (11.0%)	18 (10.3%)	18 (11.8%)	
Previously Tested Positive	121 (36.9%)	0.45 (0.22)	0.41 (0.21)	0.107
Work in healthcare	37 (11.3%)	67 (38.3%)	54 (35.3%)	0.575
Work in high-risk setting	33 (10.1%)	22 (12.6%)	15 (9.8%)	0.429
Live in high-risk setting	16 (4.9%)	21 (12.0%)	12 (7.8%)	0.212
Social vulnerability index [b], Mean (SD)	0.43 (0.22)	11 (6.3%)	5 (3.3%)	0.206
Self-Reported Comorbidity				
Number of comorbidities, Mean (SD)	0.35 (0.65)	0.45 (0.72)	0.24 (0.55)	0.003
Asthma or Chronic Lung Disease	30 (9.2%)	23 (13.1%)	7 (4.6%)	0.007
Cirrhosis of the liver	1 (0.3%)	1 (0.6%)	0 (0.0%)	0.349
Immunocompromised Conditions or Weakened Immune System [c]	16 (4.9%)	9 (5.1%)	7 (4.6%)	0.812
Diabetes	11 (3.4%)	8 (4.6%)	3 (2.0%)	0.190
Heart Conditions or Hypertension	41 (12.5%)	27 (15.4%)	14 (9.2%)	0.086
Overweight or obesity	16 (4.9%)	11 (6.3%)	5 (3.3%)	0.206
At least 1 comorbidity	87 (26.5%)	59 (33.7%)	28 (18.3%)	0.002

	All	With Long COVID	Without Long COVID	*p*-Value
Index day [d] acute COVID-19 symptoms				
Number of acute COVID-19 symptoms, Mean (SD)	5.39 (2.57)	5.91 (2.41)	4.80 (2.62)	0.000
Systemic symptoms				
Fever	127 (38.7%)	69 (39.4%)	58 (37.9%)	0.778
Chills	165 (50.3%)	103 (58.9%)	62 (40.5%)	0.001
Muscle or Body Aches	183 (55.8%)	107 (61.1%)	76 (49.7%)	0.037
Headache	224 (68.3%)	131 (74.9%)	93 (60.8%)	0.006
Fatigue	204 (62.2%)	119 (68.0%)	85 (55.6%)	0.020
Respiratory symptoms				
Shortness of Breath or Difficulty Breathing	42 (12.8%)	32 (18.3%)	10 (6.5%)	0.002
Cough	243 (74.1%)	134 (76.6%)	109 (71.2%)	0.272
Sore Throat	187 (57.0%)	106 (60.6%)	81 (52.9%)	0.164
New/Recent Loss of Taste or Smell	35 (10.7%)	22 (12.6%)	13 (8.5%)	0.233
Congestion or Runny Nose	247 (75.3%)	143 (81.7%)	104 (68.0%)	0.004
GI symptoms				
Nausea or Vomiting	42 (12.8%)	24 (13.7%)	18 (11.8%)	0.598
Diarrhea	69 (21.0%)	44 (25.1%)	25 (16.3%)	0.051

SD: Standard Deviation

[a] Definitions in Di Fusco et al (2023) [6]

[b] The Social Vulnerability Index uses 16 U.S. census variables to help local officials identify communities that may need support before, during, or after disasters.

[c] Immunocompromised conditions include compromised immune system (such as from immuno-compromising drugs, solid organ or blood stem cell transplant, HIV, or other conditions), conditions that result in a weakened immune system, including cancer treatment, and kidney failure or end stage renal disease

[d] COVID-19 test nasal swab day

Supplemental Table S2. Summary of HRQoL and WPAI results for those with long COVID and those without long COVID, with base case long COVID definition (≥ 3 symptoms)

	**With long COVID Number of post-COVID symptoms ≥3**					**Without long COVID Number of post-COVID symptoms <3**					**Between cohort difference: ≥3 vs. <3**		
	**Mean Score**		**Mean Change from pre-COVID-19**			**Mean Score**		**Mean Change from pre-COVID-19**					
	**n**	**Mean (SD)**	**Mean (SD)**	***p*-Value**	**ES**	**n**	**Mean (SD)**	**Mean (SD)**	***p*-Value**	**ES**	**Mean (SD)**	***p*-Value**	**ES**
EQ VAS													
Pre-COVID	130	84.9 (12.2)				198	88.6 (10.2)				-3.7 (11.0)	0.004	-0.33
Day 3	127	66.9 (18.1)	-18.1 (16.3)	0.000	-1.11	198	77.5 (16.1)	77.5 (16.1)	0.000	-0.92	-10.6 (16.9)	0.000	-0.63
Week 4	123	74.2 (16.2)	-10.9 (15.6)	0.000	-0.70	197	86.7 (11.4)	86.7 (11.4)	0.001	-0.23	-12.5 (13.5)	0.000	-0.93
Month 3	108	76.4 (16.0)	-9.0 (15.2)	0.000	-0.59	182	86.3 (12.0)	86.3 (12.0)	0.001	-0.26	-9.9 (13.7)	0.000	-0.72
Month 6	89	74.5 (17.2)	-10.0 (16.6)	0.000	-0.60	168	87.6 (11.2)	87.6 (11.2)	0.015	-0.19	-13.1 (13.6)	0.000	-0.96
EQ-5D-5L Utility Index (U.S. weights)													
Pre-COVID	130	0.88 (0.14)				198	0.94 (0.10)				-0.06 (0.12)	0.000	-0.50
Day 3	130	0.66 (0.26)	-0.22 (0.24)	0.000	-0.91	198	0.87 (0.15)	0.87 (0.15)	0.000	-0.63	-0.21 (0.20)	0.000	-1.04
Week 4	130	0.74 (0.20)	-0.14 (0.16)	0.000	-0.85	198	0.94 (0.10)	0.94 (0.10)	0.623	-0.03	-0.19 (0.14)	0.000	-1.33
Month 3	109	0.74 (0.24)	-0.15 (0.19)	0.000	-0.82	183	0.92 (0.13)	0.92 (0.13)	0.076	-0.13	-0.18 (0.18)	0.000	-1.02
Month 6	91	0.73 (0.24)	-0.16 (0.21)	0.000	-0.73	169	0.93 (0.12)	0.93 (0.12)	0.032	-0.17	-0.20 (0.17)	0.000	-1.16
WPAI GH													
Absenteeism													

Pre-COVID	94	7.6 (21.2)				151	7.4 (21.6)				0.2 (21.4)	0.943	0.01
Week 1	92	66.5 (36.3)	60.7 (38.1)	0.000	1.59	151	49.0 (38.8)	49.0 (38.8)	0.000	1.10	17.5 (37.9)	0.001	0.46
Week 4	86	6.2 (16.1)	-1.5 (24.7)	0.589	-0.06	146	2.7 (14.6)	2.7 (14.6)	0.088	- 0.15	3.5 (15.2)	0.087	0.23
Month 3	78	9.6 (23.4)	1.4 (33.2)	0.717	0.04	134	3.8 (13.1)	3.8 (13.1)	0.272	- 0.10	5.8 (17.6)	0.023	0.33
Month 6	61	8.8 (19.0)	-0.7 (30.4)	0.856	-0.02	126	3.1 (14.2)	3.1 (14.2)	0.094	- 0.16	5.7 (15.9)	0.023	0.36
Presenteeism													
Pre-COVID	92	13.2 (22.6)				147	7.6 (16.5)				5.6 (19.1)	0.028	0.29
Week 1	59	56.1 (25.8)	40.7 (31.6)	0.000	1.29	117	39.6 (30.1)	39.6 (30.1)	0.000	1.04	16.5 (28.7)	0.000	0.58
Week 4	85	29.1 (23.4)	13.9 (29.1)	0.000	0.48	143	6.6 (14.7)	6.6 (14.7)	0.702	0.03	22.4 (18.4)	0.000	1.22
Month 3	75	23.2 (24.7)	12.8 (30.8)	0.001	0.42	133	9.3 (20.7)	9.3 (20.7)	0.640	0.04	13.9 (22.2)	0.000	0.62
Month 6	60	29.7 (23.9)	18.1 (34.4)	0.000	0.53	124	6.5 (13.9)	6.5 (13.9)	0.398	- 0.08	23.1 (17.8)	0.000	1.30
Work productivity loss													
Pre-COVID	91	15.1 (24.7)				147	11.5 (21.5)				3.6 (22.8)	0.236	0.16
Week 1	59	74.7 (23.3)	56.9 (31.8)	0.000	1.79	117	56.0 (33.0)	56.0 (33.0)	0.000	1.31	18.7 (30.1)	0.000	0.62
Week 4	85	31.7 (25.4)	15.1 (32.8)	0.000	0.46	143	7.0 (15.0)	7.0 (15.0)	0.312	- 0.09	24.7 (19.5)	0.000	1.27
Month 3	74	26.1 (26.5)	13.3 (35.2)	0.003	0.38	133	11.3 (23.1)	11.3 (23.1)	0.746	- 0.03	14.8 (24.4)	0.000	0.61
Month 6	60	33.9 (26.2)	19.9 (38.0)	0.000	0.52	124	7.7 (15.8)	7.7 (15.8)	0.084	- 0.17	26.2 (19.8)	0.000	1.32
Activity impairment													

Pre-COVID	130	21.3 (29.3)				198	11.1 (20.8)				10.2 (24.5)	0.000	0.42
Week 1	130	65.5 (25.1)	44.2 (36.2)	0.000	1.22	198	42.2 (31.3)	42.2 (31.3)	0.000	0.96	23.4 (29.0)	0.000	0.80
Week 4	130	38.1 (25.7)	16.8 (35.6)	0.000	0.47	198	9.0 (18.0)	9.0 (18.0)	0.215	- 0.09	29.0 (21.3)	0.000	1.36
Month 3	109	31.7 (28.3)	12.9 (36.6)	0.000	0.35	183	12.3 (21.5)	12.3 (21.5)	0.976	0.00	19.4 (24.3)	0.000	0.80
Month 6	91	37.5 (27.5)	17.4 (39.2)	0.000	0.44	169	8.4 (17.2)	8.4 (17.2)	0.108	- 0.12	29.1 (21.3)	0.000	1.36
Hours missed due to health													
Pre-COVID	95	4.7 (15.1)				153	3.9 (11.8)				0.8 (13.2)	0.654	0.06
Week 1	94	28.6 (20.2)	24.3 (20.7)	0.000	1.18	153	19.3 (16.5)	19.3 (16.5)	0.000	0.91	9.2 (18.0)	0.000	0.51
Week 4	89	2.6 (7.7)	-2.3 (16.5)	0.205	-0.14	150	0.7 (4.2)	0.7 (4.2)	0.018	- 0.20	1.9 (5.7)	0.012	0.34
Month 3	81	3.4 (8.9)	-1.7 (18.0)	0.418	-0.09	140	1.7 (6.9)	1.7 (6.9)	0.059	- 0.17	1.7 (7.7)	0.113	0.22
Month 6	66	2.8 (6.1)	-2.7 (16.3)	0.208	-0.17	138	1.3 (6.4)	1.3 (6.4)	0.054	- 0.18	1.5 (6.3)	0.126	0.23
Actual hours worked													
Pre-COVID	95	36.7 (13.5)				153	37.8 (14.6)				-1.1 (14.2)	0.557	-0.08
Week 1	93	14.3 (16.6)	-22.8 (17.4)	0.000	-1.31	152	21.8 (17.9)	21.8 (17.9)	0.000	- 0.83	-7.5 (17.4)	0.001	-0.43
Week 4	89	34.1 (14.7)	-2.3 (16.3)	0.195	-0.14	150	35.8 (13.8)	35.8 (13.8)	0.407	- 0.07	-1.7 (14.2)	0.371	-0.12
Month 3	82	34.8 (15.1)	-1.6 (20.8)	0.504	-0.08	141	35.1 (14.6)	35.1 (14.6)	0.059	- 0.17	-0.3 (14.8)	0.873	-0.02
Month 6	66	31.1 (15.7)	-4.4 (17.1)	0.055	-0.25	138	33.4 (15.9)	33.4 (15.9)	0.087	- 0.16	-2.3 (15.8)	0.334	-0.15

Supplemental Table S3. Summary of HRQoL and WPAI results for those with long COVID and those without long COVID, with sensitivity analysis long COVID definition (≥ 2 symptoms)

	**With long COVID Number of post-COVID symptoms ≥2**					**Without long COVID Number of post-COVID symptoms <2**					**Between cohort difference: ≥2 vs. <2**		
	**Mean Score**		**Mean Change from pre-COVID-19**			**Mean Score**		**Mean Change from pre-COVID-19**					
	**n**	**Mean (SD)**	**Mean (SD)**	***p*-Value**	**ES**	**n**	**Mean (SD)**	**Mean (SD)**	***p*-Value**	**ES**	**Mean (SD)**	***p*-Value**	**ES**
EQ VAS													
Pre-COVID	175	85.1 (11.8)				153	89.4 (9.9)				-4.3 (11.0)	0.001	-0.39
Day 3	172	68.7 (18.0)	-16.5 (15.3)	0.000	-1.08	153	78.6 (15.8)	78.6 (15.8)	0.000	-0.87	-9.9 (17.0)	0.000	-0.58
Week 4	168	76.4 (15.8)	-8.9 (14.3)	0.000	-0.62	152	87.9 (10.7)	87.9 (10.7)	0.024	-0.19	-11.5 (13.6)	0.000	-0.84
Month 3	136	76.8 (15.9)	-8.4 (14.1)	0.000	-0.60	154	87.8 (10.8)	87.8 (10.8)	0.025	-0.18	-11.0 (13.4)	0.000	-0.82
Month 6	113	76.1 (16.3)	-8.6 (15.3)	0.000	-0.56	144	88.6 (11.0)	88.6 (11.0)	0.079	-0.15	-12.5 (13.6)	0.000	-0.92
EQ-5D-5L Utility Index (U.S. weights)													
Pre-COVID	175	0.89 (0.14)				153	0.95 (0.09)				-0.06 (0.12)	0.000	-0.46
Day 3	175	0.70 (0.25)	-0.19 (0.22)	0.000	-0.85	153	0.88 (0.14)	0.88 (0.14)	0.000	-0.59	-0.19 (0.20)	0.000	-0.92
Week 4	175	0.79 (0.19)	-0.10 (0.16)	0.000	-0.66	153	0.95 (0.09)	0.95 (0.09)	0.888	-0.01	-0.16 (0.15)	0.000	-1.03
Month 3	137	0.77 (0.22)	-0.13 (0.18)	0.000	-0.76	155	0.93 (0.14)	0.93 (0.14)	0.388	-0.07	-0.16 (0.18)	0.000	-0.90
Month 6	115	0.75 (0.23)	-0.14 (0.20)	0.000	-0.71	145	0.94 (0.12)	0.94 (0.12)	0.449	-0.06	-0.18 (0.18)	0.000	-1.05
WPAI GH													

Absenteeism													
Pre-COVID	135	7.7 (22.0)				110	7.1 (20.7)				0.7 (21.4)	0.807	0.03
Week 1	133	62.4 (38.0)	55.8 (38.8)	0.000	1.44	110	47.5 (38.3)	47.5 (38.3)	0.000	1.06	15.0 (38.1)	0.003	0.39
Week 4	127	5.2 (16.0)	-2.5 (26.4)	0.290	-0.10	105	2.6 (14.2)	2.6 (14.2)	0.167	- 0.14	2.7 (15.2)	0.187	0.17
Month 3	99	9.4 (22.2)	1.4 (32.0)	0.668	0.04	113	2.9 (12.1)	2.9 (12.1)	0.169	- 0.14	6.4 (17.6)	0.008	0.37
Month 6	81	7.5 (17.6)	-1.2 (28.1)	0.718	-0.04	106	3.0 (14.6)	3.0 (14.6)	0.113	- 0.17	4.4 (16.0)	0.061	0.28
Presenteeism													
Pre-COVID	131	10.8 (20.1)				108	8.4 (18.1)				2.3 (19.2)	0.350	0.12
Week 1	88	52.0 (26.6)	40.1 (30.3)	0.000	1.33	88	38.2 (31.1)	38.2 (31.1)	0.000	0.95	13.9 (28.9)	0.002	0.48
Week 4	125	22.8 (22.4)	11.3 (25.8)	0.000	0.44	103	5.5 (15.4)	5.5 (15.4)	0.573	- 0.06	17.3 (19.6)	0.000	0.88
Month 3	96	21.6 (24.4)	11.8 (30.3)	0.000	0.39	112	8.1 (20.2)	8.1 (20.2)	0.857	- 0.02	13.4 (22.2)	0.000	0.61
Month 6	80	26.0 (23.4)	12.3 (33.2)	0.003	0.37	104	4.9 (12.5)	4.9 (12.5)	0.600	- 0.06	21.1 (18.0)	0.000	1.17
Work productivity loss													
Pre-COVID	130	13.1 (22.4)				108	12.6 (23.4)				0.5 (22.8)	0.867	0.02
Week 1	88	69.4 (26.8)	55.8 (32.0)	0.000	1.74	88	55.1 (33.9)	55.1 (33.9)	0.000	1.22	14.3 (30.6)	0.002	0.47
Week 4	125	24.7 (24.3)	11.3 (29.8)	0.000	0.38	103	5.8 (15.6)	5.8 (15.6)	0.113	- 0.16	18.9 (20.9)	0.000	0.91
Month 3	95	24.8 (26.8)	12.9 (34.4)	0.001	0.37	112	9.6 (21.8)	9.6 (21.8)	0.252	- 0.11	15.2 (24.2)	0.000	0.63
Month 6	80	29.8 (25.9)	13.8 (37.0)	0.003	0.37	104	5.8 (13.9)	5.8 (13.9)	0.098	- 0.18	24.1 (20.0)	0.000	1.20
Activity impairment													

Pre-COVID	175	18.9 (28.3)				153	10.8 (19.7)				8.1 (24.7)	0.003	0.33
Week 1	175	62.0 (26.4)	43.1 (34.9)	0.000	1.24	153	39.3 (31.9)	39.3 (31.9)	0.000	0.88	22.7 (29.1)	0.000	0.78
Week 4	175	31.3 (26.1)	12.3 (34.0)	0.000	0.36	153	8.3 (18.7)	8.3 (18.7)	0.172	-0.11	23.0 (22.9)	0.000	1.00
Month 3	137	30.0 (28.0)	11.2 (36.2)	0.000	0.31	155	10.3 (20.0)	10.3 (20.0)	0.617	-0.04	19.7 (24.1)	0.000	0.82
Month 6	115	33.4 (27.0)	13.0 (37.9)	0.000	0.34	145	6.8 (16.4)	6.8 (16.4)	0.147	-0.12	26.6 (21.8)	0.000	1.22
Hours missed due to health													
Pre-COVID	136	4.4 (13.9)				112	3.9 (12.2)				0.5 (13.2)	0.769	0.04
Week 1	136	26.1 (19.6)	22.1 (19.8)	0.000	1.11	111	18.9 (16.4)	18.9 (16.4)	0.000	0.88	7.2 (18.2)	0.002	0.39
Week 4	130	2.1 (6.9)	-2.4 (15.2)	0.078	-0.16	109	0.6 (4.0)	0.6 (4.0)	0.057	-0.19	1.5 (5.8)	0.040	0.27
Month 3	104	3.5 (9.4)	-1.1 (16.4)	0.518	-0.07	117	1.2 (5.8)	1.2 (5.8)	0.035	-0.21	2.3 (7.7)	0.026	0.30
Month 6	86	2.8 (7.5)	-1.9 (15.7)	0.280	-0.12	118	1.1 (5.2)	1.1 (5.2)	0.029	-0.22	1.8 (6.3)	0.051	0.28
Actual hours worked													
Pre-COVID	136	37.0 (13.0)				112	37.9 (15.6)				-0.9 (14.2)	0.637	-0.06
Week 1	135	15.6 (16.7)	-21.5 (16.9)	0.000	-1.27	110	23.0 (18.2)	23.0 (18.2)	0.000	-0.73	-7.4 (17.4)	0.001	-0.43
Week 4	130	34.5 (14.1)	-2.1 (15.1)	0.115	-0.14	109	36.0 (14.2)	36.0 (14.2)	0.651	-0.04	-1.6 (14.2)	0.398	-0.11
Month 3	105	34.3 (15.4)	-1.9 (19.9)	0.339	-0.10	118	35.6 (14.1)	35.6 (14.1)	0.096	-0.16	-1.3 (14.8)	0.503	-0.09
Month 6	86	32.9 (15.1)	-2.4 (16.0)	0.189	-0.15	118	32.4 (16.4)	32.4 (16.4)	0.031	-0.21	0.5 (15.9)	0.807	0.03

Supplemental Table S4. Least-Square Estimates of HRQoL and WPAI results for those with long COVID and those without long COVID, with sensitivity analysis long COVID definition (≥ 2 symptoms)

	**With long COVID Number of post-COVID symptoms ≥2**				**Without long COVID Number of post-COVID symptoms <2**				**Between Cohort Difference: ≥2 vs. <2**		
	**Mean Score**	**Mean Change from pre-COVID**			**Mean Score**	**Mean Change from pre-COVID**					
	**LSE (95% CI)**	**LSE (95% CI)**	***p*-Value**	**ES**	**LSE (95% CI)**	**LSE (95% CI)**	***p*-Value**	**ES**	**LSE (95% CI)**	***p*-Value**	**ES**
EQ VAS											
Pre-COVID	85.1 (11.8)				89.4 (9.9)				-4.3 (11.0)	0.001	-0.39
Day 3	71.9 (69.0, 74.9)	-15.4 (-18.4, -12.5)	0.000	-1.01	77.8 (74.8, 80.9)	-9.5 (-12.6, -6.5)	0.000	-0.77	-5.9 (-9.0, -2.8)	0.000	-0.35
Week 4	80.5 (77.9, 83.1)	-6.9 (-9.4, -4.3)	0.000	-0.48	86.6 (83.9, 89.2)	-0.8 (-3.4, 1.8)	0.552	-0.10	-6.1 (-8.2, -3.9)	0.000	-0.44
Month 3	80.9 (78.2, 83.6)	-6.4 (-9.1, -3.8)	0.000	-0.46	86.4 (83.8, 89.0)	-0.9 (-3.5, 1.6)	0.474	-0.10	-5.5 (-7.7, -3.3)	0.000	-0.41
Month 6	80.4 (77.5, 83.3)	-7.0 (-9.9, -4.1)	0.000	-0.46	86.9 (84.2, 89.6)	-0.5 (-3.2, 2.2)	0.716	-0.06	-6.5 (-9.1, -3.8)	0.000	-0.48
EQ-5D-5L Utility Index (U.S. weights)											
Pre-COVID	0.89 (0.14)				0.95 (0.09)				-0.06 (0.12)	0.000	-0.46
Day 3	0.75 (0.71, 0.79)	-0.17 (-0.21, -0.13)	0.000	-0.75	0.87 (0.83, 0.90)	-0.05 (-0.09, -0.02)	0.006	-0.51	-0.12 (-0.15, -0.08)	0.000	-0.57
Week 4	0.84 (0.81, 0.87)	-0.08 (-0.11, -0.05)	0.000	-0.50	0.92 (0.89, 0.95)	0.00 (-0.03, 0.03)	0.937	-0.01	-0.08 (-0.10, -0.05)	0.000	-0.50
Month 3	0.84 (0.81, 0.88)	-0.08 (-0.11, -0.04)	0.000	-0.43	0.90 (0.86, 0.93)	-0.02 (-0.06, 0.01)	0.185	-0.18	-0.05 (-0.08, -0.03)	0.000	-0.30
Month 6	0.83 (0.79, 0.86)	-0.09 (-0.13, -0.06)	0.000	-0.47	0.90 (0.87, 0.93)	-0.02 (-0.05, 0.01)	0.270	-0.20	-0.07 (-0.10, -0.05)	0.000	-0.43
WPAI GH											
Absenteeism											
Pre-COVID	7.7 (22.0)				7.1 (20.7)				0.7 (21.4)	0.807	0.03
Week 1	59.5 (52.4, 66.6)	52.6 (45.5, 59.7)	0.000	1.36	47.9 (40.2, 55.6)	41.0 (33.3, 48.7)	0.000	1.06	11.6 (2.3, 20.8)	0.015	0.30
Week 4	5.0 (1.0, 9.0)	-1.9 (-5.9, 2.1)	0.357	-0.07	4.1 (-0.1, 8.4)	-2.7 (-7.0, 1.5)	0.206	-0.12	0.8 (-2.3, 4.0)	0.595	0.06
Month 3	8.6 (4.0, 13.2)	1.8 (-2.8, 6.4)	0.449	0.06	6.6 (1.9, 11.2)	-0.3 (-5.0, 4.3)	0.896	-0.01	2.1 (-1.8, 6.0)	0.296	0.12

Month 6	7.3 (2.6, 12.0)	0.4 (-4.3, 5.1)	0.866	0.01	3.9 (-0.7, 8.5)	-3.0 (-7.5, 1.6)	0.203	-0.14	3.4 (-1.1, 7.8)	0.136	0.21
Presenteeism											
Pre-COVID	10.8 (20.1)				8.4 (18.1)				2.3 (19.2)	0.350	0.12
Week 1	46.3 (38.8, 53.8)	36.7 (29.2, 44.2)	0.000	1.21	37.5 (29.9, 45.2)	27.9 (20.2, 35.5)	0.000	0.85	8.8 (0.4, 17.2)	0.041	0.30
Week 4	14.7 (9.1, 20.3)	5.0 (-0.6, 10.6)	0.079	0.19	3.1 (-2.8, 8.9)	-6.6 (-12.5, -0.7)	0.028	-0.31	11.6 (6.9, 16.3)	0.000	0.59
Month 3	14.3 (7.6, 20.9)	4.6 (-2.0, 11.2)	0.172	0.15	7.5 (1.2, 13.8)	-2.1 (-8.4, 4.2)	0.508	-0.10	6.7 (0.6, 12.8)	0.031	0.30
Month 6	18.5 (12.1, 25.0)	8.9 (2.4, 15.3)	0.007	0.27	4.3 (-1.9, 10.4)	-5.4 (-11.5, 0.7)	0.085	-0.27	14.3 (8.3, 20.2)	0.000	0.79
Work productivity loss											
Pre-COVID	13.1 (22.4)				12.6 (23.4)				0.5 (22.8)	0.867	0.02
Week 1	63.2 (55.1, 71.3)	51.0 (43.0, 59.1)	0.000	1.60	54.9 (46.8, 63.1)	42.8 (34.6, 50.9)	0.000	1.15	8.3 (-0.5, 17.1)	0.064	0.27
Week 4	16.7 (10.6, 22.9)	4.6 (-1.6, 10.7)	0.147	0.15	3.6 (-2.9, 10.0)	-8.6 (-15.0, -2.2)	0.009	-0.33	13.2 (8.1, 18.2)	0.000	0.63
Month 3	16.8 (9.5, 24.1)	4.6 (-2.7, 11.9)	0.215	0.13	9.8 (2.9, 16.8)	-2.3 (-9.3, 4.6)	0.509	-0.09	6.9 (0.3, 13.6)	0.042	0.29
Month 6	21.4 (14.4, 28.5)	9.3 (2.2, 16.3)	0.010	0.25	5.9 (-0.8, 12.6)	-6.2 (-12.9, 0.5)	0.068	-0.25	15.5 (9.2, 21.8)	0.000	0.77
Activity impairment											
Pre-COVID	18.9 (28.3)				10.8 (19.7)				8.1 (24.7)	0.003	0.33
Week 1	54.3 (48.7, 59.9)	39.4 (33.8, 45.1)	0.000	1.13	38.0 (32.2, 43.8)	23.1 (17.3, 28.9)	0.000	0.71	16.4 (10.2, 22.5)	0.000	0.56
Week 4	22.0 (17.2, 26.9)	7.2 (2.3, 12.0)	0.004	0.21	7.3 (2.4, 12.2)	-7.6 (-12.5, -2.7)	0.003	-0.33	14.7 (10.4, 19.1)	0.000	0.64
Month 3	20.8 (15.4, 26.1)	5.9 (0.5, 11.2)	0.031	0.16	11.5 (6.4, 16.6)	-3.4 (-8.5, 1.7)	0.191	-0.15	9.3 (4.3, 14.2)	0.000	0.38
Month 6	23.8 (18.4, 29.2)	8.9 (3.5, 14.4)	0.001	0.24	7.5 (2.5, 12.5)	-7.4 (-12.4, -2.4)	0.004	-0.34	16.3 (11.2, 21.5)	0.000	0.75
Hours missed due to health											
Pre-COVID	4.4 (13.9)				3.9 (12.2)				0.5 (13.2)	0.769	0.04
Week 1	24.0 (20.8, 27.3)	20.1 (16.9, 23.3)	0.000	1.01	17.7 (14.2, 21.2)	13.7 (10.2, 17.2)	0.000	0.78	6.4 (2.1, 10.6)	0.004	0.35
Week 4	1.5 (-0.2, 3.1)	-2.5 (-4.1, -0.8)	0.004	-0.16	0.5 (-1.2, 2.2)	-3.4 (-5.2, -1.7)	0.000	-0.31	1.0 (-0.5, 2.4)	0.181	0.17
Month 3	2.2 (0.2, 4.2)	-1.7 (-3.7, 0.3)	0.095	-0.10	1.6 (-0.4, 3.5)	-2.4 (-4.3, -0.4)	0.018	-0.17	0.6 (-1.3, 2.6)	0.517	0.08
Month 6	1.6 (-0.3, 3.6)	-2.3 (-4.2, -0.4)	0.019	-0.15	0.8 (-0.9, 2.6)	-3.1 (-4.9, -1.3)	0.001	-0.25	0.8 (-1.1, 2.6)	0.405	0.12

Actual hours worked											
Pre-COVID	37.0 (13.0)				37.9 (15.6)				-0.9 (14.2)	0.637	-0.06
Week 1	16.9 (13.1, 20.8)	-20.3 (-24.2, -16.5)	0.000	-1.20	22.7 (18.6, 26.8)	-14.6 (-18.7, -10.5)	0.000	-0.76	-5.8 (-9.8, -1.8)	0.005	-0.33
Week 4	34.7 (31.2, 38.2)	-2.6 (-6.1, 0.9)	0.147	-0.17	35.5 (31.8, 39.2)	-1.8 (-5.5, 1.9)	0.333	-0.13	-0.8 (-4.0, 2.5)	0.648	-0.05
Month 3	34.1 (30.0, 38.2)	-3.2 (-7.3, 0.9)	0.124	-0.16	34.0 (30.1, 37.9)	-3.2 (-7.2, 0.7)	0.102	-0.20	0.1 (-3.9, 4.0)	0.978	0.00
Month 6	34.0 (29.7, 38.3)	-3.3 (-7.6, 1.0)	0.137	-0.21	32.0 (28.1, 36.0)	-5.3 (-9.2, -1.3)	0.009	-0.30	2.0 (-2.3, 6.3)	0.365	0.13

Supplemental Table S5. Summary of HRQoL and WPAI results for patients age ≥50 and age<50 years

	Age≥50 years					Age<50 years					Age≥50 vs. <50 years		
	Mean Score		Mean Change from pre-COVID			Mean Score		Mean Change from pre-COVID					
	n	Mean (SD)	Mean (SD)	*p*-Value	ES	n	Mean (SD)	Mean (SD)	*p*-Value	ES	Mean (SD)	*p*-Value	ES
EQ VAS													
Pre-COVID	95	86.1 (12.7)				233	87.6 (10.5)				-1.5 (11.2)	0.282	-0.13
Day 3	94	73.6 (19.2)	-12.6 (14.9)	0.000	-0.85	231	73.2 (17.1)	73.2 (17.1)	0.000	-1.02	0.4 (17.7)	0.848	0.02
Week 4	93	80.2 (15.7)	-6.1 (13.8)	0.000	-0.44	227	82.6 (14.4)	82.6 (14.4)	0.000	-0.43	-2.4 (14.8)	0.194	-0.16
Month 3	81	81.0 (14.7)	-5.5 (12.0)	0.000	-0.46	209	83.3 (14.4)	83.3 (14.4)	0.000	-0.37	-2.3 (14.4)	0.228	-0.16
Month 6	70	82.8 (15.3)	-3.9 (13.1)	0.015	-0.30	187	83.2 (14.8)	83.2 (14.8)	0.000	-0.38	-0.3 (15.0)	0.870	-0.02
EQ-5D-5L Utility Index (U.S. weights)													
Pre-COVID	95	0.92 (0.12)				233	0.92 (0.12)				0.00 (0.12)	0.864	0.02
Day 3	95	0.80 (0.21)	-0.11 (0.15)	0.000	-0.75	233	0.78 (0.23)	0.78 (0.23)	0.000	-0.68	0.03 (0.22)	0.332	0.12
Week 4	95	0.83 (0.19)	-0.09 (0.13)	0.000	-0.69	233	0.87 (0.17)	0.87 (0.17)	0.000	-0.30	-0.04 (0.17)	0.038	-0.25
Month 3	81	0.82 (0.24)	-0.10 (0.17)	0.000	-0.60	211	0.87 (0.18)	0.87 (0.18)	0.000	-0.34	-0.05 (0.20)	0.081	-0.23
Month 6	71	0.84 (0.21)	-0.09 (0.15)	0.000	-0.60	189	0.86 (0.19)	0.86 (0.19)	0.000	-0.34	-0.02 (0.20)	0.494	-0.10
WPAI GH													
Absenteeism													
Pre-COVID	55	8.0 (20.5)				190	7.3 (21.7)				0.7 (21.4)	0.821	0.03

Week 1	54	62.2 (39.1)	55.3 (38.7)	0.000	1.43	189	53.8 (38.6)	53.8 (38.6)	0.000	1.21	8.4 (38.7)	0.159	0.22
Week 4	53	7.6 (24.3)	0.8 (27.5)	0.835	0.03	179	3.0 (11.1)	3.0 (11.1)	0.035	-0.16	4.7 (15.1)	0.050	0.31
Month 3	45	6.2 (21.1)	1.8 (22.2)	0.616	0.08	167	5.9 (16.9)	5.9 (16.9)	0.477	-0.06	0.4 (17.9)	0.905	0.02
Month 6	45	3.0 (10.7)	-2.0 (18.4)	0.511	-0.11	142	5.6 (17.4)	5.6 (17.4)	0.249	-0.10	-2.6 (16.1)	0.354	-0.16
Presenteeism													
Pre-COVID	54	10.7 (22.1)				185	9.4 (18.3)				1.3 (19.2)	0.654	0.07
Week 1	36	43.3 (32.8)	36.8 (32.3)	0.000	1.14	140	45.6 (28.9)	45.6 (28.9)	0.000	1.11	-2.2 (29.8)	0.688	-0.08
Week 4	50	13.8 (18.4)	4.7 (22.2)	0.166	0.21	178	15.3 (22.1)	15.3 (22.1)	0.003	0.23	-1.5 (21.4)	0.654	-0.07
Month 3	43	13.5 (20.7)	2.4 (19.8)	0.465	0.12	165	14.5 (23.8)	14.5 (23.8)	0.010	0.21	-1.1 (23.2)	0.790	-0.05
Month 6	45	13.6 (16.9)	2.2 (27.0)	0.629	0.08	139	14.2 (22.0)	14.2 (22.0)	0.027	0.20	-0.7 (20.9)	0.848	-0.03
Work productivity loss													
Pre-COVID	54	16.0 (25.9)				184	12.0 (21.8)				4.0 (22.8)	0.257	0.18
Week 1	36	62.0 (34.6)	52.2 (35.6)	0.000	1.46	140	62.3 (30.6)	62.3 (30.6)	0.000	1.43	-0.4 (31.4)	0.951	-0.01
Week 4	50	14.9 (20.5)	2.5 (27.3)	0.540	0.09	178	16.6 (23.5)	16.6 (23.5)	0.042	0.16	-1.6 (22.9)	0.654	-0.07
Month 3	43	14.6 (21.9)	-0.3 (22.3)	0.925	-0.02	164	17.1 (26.2)	17.1 (26.2)	0.057	0.16	-2.5 (25.4)	0.564	-0.10
Month 6	45	16.3 (18.8)	1.0 (30.8)	0.841	0.03	139	16.2 (24.6)	16.2 (24.6)	0.161	0.13	0.1 (23.3)	0.984	0.00
Activity impairment													
Pre-COVID	95	17.5 (26.7)				233	14.2 (24.2)				3.3 (25.0)	0.283	0.13
Week 1	95	51.6 (32.1)	34.1 (33.7)	0.000	1.01	233	51.4 (30.9)	51.4 (30.9)	0.000	1.07	0.2 (31.2)	0.957	0.01
Week 4	95	24.7 (27.1)	7.3 (32.3)	0.031	0.22	233	18.8 (24.9)	18.8 (24.9)	0.017	0.16	5.9 (25.5)	0.059	0.23

Month 3	81	25.6 (25.7)	9.8 (25.3)	0.001	0.39	211	17.3 (25.8)	17.3 (25.8)	0.189	0.09	8.3 (25.8)	0.014	0.32
Month 6	71	21.3 (24.8)	5.6 (30.6)	0.126	0.18	189	17.6 (25.6)	17.6 (25.6)	0.097	0.12	3.7 (25.4)	0.296	0.15
Hours missed due to health													
Pre-COVID	57	5.4 (13.5)				191	3.9 (13.1)				1.5 (13.2)	0.460	0.11
Week 1	56	25.6 (21.1)	20.8 (23.5)	0.000	0.88	191	22.1 (17.7)	22.1 (17.7)	0.000	1.05	3.5 (18.5)	0.217	0.19
Week 4	53	2.2 (8.1)	-2.5 (14.0)	0.217	-0.18	186	1.2 (5.0)	1.2 (5.0)	0.028	-0.17	1.1 (5.8)	0.235	0.19
Month 3	49	1.6 (6.0)	-1.9 (9.8)	0.198	-0.19	172	2.5 (8.2)	2.5 (8.2)	0.119	-0.12	-0.9 (7.7)	0.483	-0.11
Month 6	47	1.9 (8.1)	-1.9 (14.7)	0.417	-0.13	157	1.8 (5.8)	1.8 (5.8)	0.031	-0.18	0.1 (6.4)	0.919	0.02
Actual hours worked													
Pre-COVID	56	39.4 (14.8)				192	36.8 (14.0)				2.5 (14.2)	0.237	0.18
Week 1	56	18.1 (19.5)	-20.8 (20.4)	0.000	-1.02	189	19.2 (17.3)	19.2 (17.3)	0.000	-0.98	-1.0 (17.8)	0.699	-0.06
Week 4	54	35.5 (15.9)	-3.7 (16.9)	0.126	-0.22	185	35.1 (13.7)	35.1 (13.7)	0.434	-0.06	0.4 (14.2)	0.838	0.03
Month 3	49	32.6 (16.6)	-7.7 (21.2)	0.021	-0.36	174	35.7 (14.1)	35.7 (14.1)	0.524	-0.05	-3.1 (14.7)	0.195	-0.21
Month 6	47	35.0 (16.0)	-2.1 (16.2)	0.428	-0.13	157	31.9 (15.8)	31.9 (15.8)	0.016	-0.20	3.1 (15.8)	0.246	0.19

Supplemental Table S6. Work hours lost at Week 1 by vaccination status

Week 1	All		Boosted [a]		Primed [a]		Unvaccinated [a]		*p*-Value
	n / Mean / Median	% / SD /Q1-Q3	n / Mean / Median	% / SD /Q1-Q3	n / Mean / Median	% / SD /Q1-Q3	n / Mean / Median	% / SD /Q1-Q3	
n, %	247	75.3%	67	77.0%	66	76.7%	114	73.5%	
Mean, SD	22.86	18.54	15.85	15.39	20.18	17.06	28.53	19.42	<0.001
Median, Q1-Q3	21	6~40	15	0~24	16	4~35	32	12~40	<0.001
Min, max	0	90	0	60	0	72	0	90	
Missing	81		20		20		41		

[a] Definitions in Di Fusco et al (2023) [6]
